# A Novel Hybrid Improved RIME Algorithm for Global Optimization Problems

**DOI:** 10.3390/biomimetics10010014

**Published:** 2024-12-31

**Authors:** Wuke Li, Xiong Yang, Yuchen Yin, Qian Wang

**Affiliations:** 1 School of Computer and Electrical Engineering, Hunan University of Arts and Science, Changde 415000, China; liwuke@huas.edu.cn; 2 Zhicheng College, Fuzhou University, Fuzhou 350002, China; 3 Teachers College, Columbia University, 525 West 120th Street, New York, NY 10027, USA; yy3243@tc.columbia.edu; 4 Department of Computer Science, Durham University, Durham DH1 3LE, UK

**Keywords:** RIME, global optimization, fitness distance balance, hybrid, metaheuristic optimization, synergistic fusion framework

## Abstract

The RIME algorithm is a novel physical-based meta-heuristic algorithm with a strong ability to solve global optimization problems and address challenges in engineering applications. It implements exploration and exploitation behaviors by constructing a rime-ice growth process. However, RIME comes with a couple of disadvantages: a limited exploratory capability, slow convergence, and inherent asymmetry between exploration and exploitation. An improved version with more efficiency and adaptability to solve these issues now comes in the form of Hybrid Estimation Rime-ice Optimization, in short, HERIME. A probabilistic model-based sampling approach of the estimated distribution algorithm is utilized to enhance the quality of the RIME population and boost its global exploration capability. A roulette-based fitness distance balanced selection strategy is used to strengthen the hard-rime phase of RIME to effectively enhance the balance between the exploitation and exploration phases of the optimization process. We validate HERIME using 41 functions from the IEEE CEC2017 and IEEE CEC2022 test suites and compare its optimization accuracy, convergence, and stability with four classical and recent metaheuristic algorithms as well as five advanced algorithms to reveal the fact that the proposed algorithm outperforms all of them. Statistical research using the Friedman test and Wilcoxon rank sum test also confirms its excellent performance. Moreover, ablation experiments validate the effectiveness of each strategy individually. Thus, the experimental results show that HERIME has better search efficiency and optimization accuracy and is effective in dealing with global optimization problems.

## 1. Introduction

Optimization is considered an essential research area in modern engineering, medicine, control science, informatics, and other fields. Its main goal is to find the optimal value of a specific performance index under certain constraints. However, with the rapid progress of AI technology, a new era has arrived, characterized by increasingly complex and diverse practical problems. They are usually characterized by multimodal, nonlinear, high-dimensional, and non-microscopic [1]. This leads to the fact that traditional optimization methods, such as gradient descent and linear programming, are insufficient for finding globally optimal solutions in most cases when trying to deal with increasingly complex optimization problems [2,3]. With the increase in computational power and the continuous development of intelligent algorithms, a category of optimization methods called metaheuristics has emerged and attracted much attention due to their excellent performance in solving complex optimization problems. Metaheuristic algorithms do not depend on a specific problem and operate in a stochastic, simple, efficient, and flexible manner [4]. They approach the problem by using random sampling within a robust solution space to obtain an initial reference estimate close to the optimal solution. This approach can be better adapted to the reality of limited processing resources. Metaheuristic algorithms have a large number of applications spanning several domains such as medical imaging [5,6], scheduling problems [7,8], feature selection [9,10], task planning [11,12], machine learning [13,14], and so on. Moreover, the no free lunch theorem [15] states that the superior performance of an algorithm in dealing with a particular optimization problem does not ensure comparability across different optimization scenarios. Therefore, the study of each meta-heuristic algorithm is of specific relevance. The NFL theorem serves as a catalyst to motivate researchers to design newer algorithms aiming to provide better solutions to different optimization problems.

**Figure 1:**
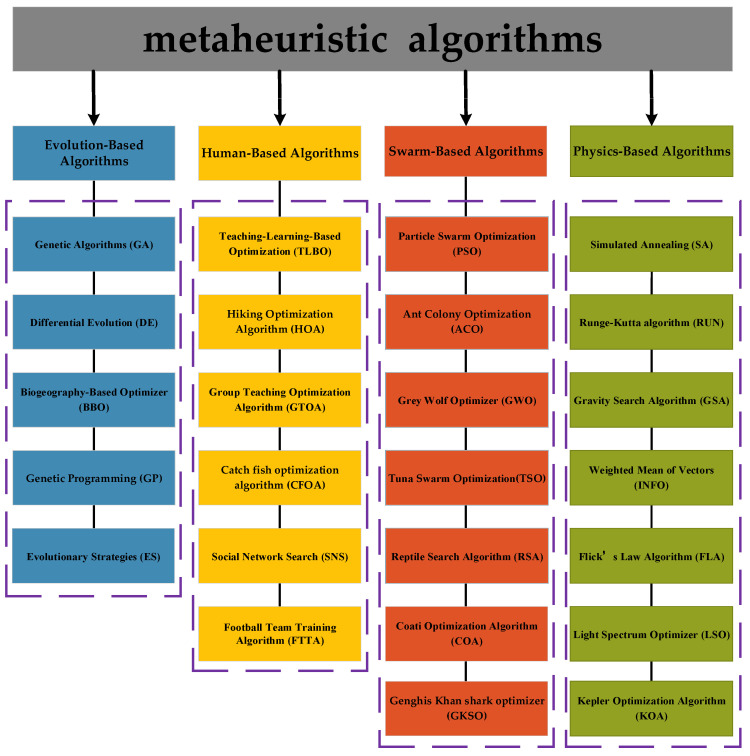
Classification of meta-heuristic optimization algorithms.

Metaheuristic algorithms are divided into four categories depending on the source of inspiration [16]: evolution-based, human-based, swarm-based, and physical-based algorithms, as illustrated in Figure 1. Evolution-based algorithms draw inspiration from selection and genetic mechanisms in nature. Genetic Algorithms (GAs) [17] and Differential Evolution (DE) [18] are typical of this category, and the rest include the Biogeography-Based Optimizer (BBO) [19], Genetic Programming (GP) [20], and Evolutionary Strategies (ES) [21]. Human-based algorithms are inspired by human behavior. Teaching–Learning-Based Optimization (TLBO) is inspired by the classroom teaching process and iteratively improves the quality of the solution by simulating the interaction between the teacher and the students [22]. Oladejo et al. proposed a Hiking Optimization Algorithm (HOA) inspired by human hiking [23]. Other human-based algorithms include Group Teaching Optimization Algorithm (GTOA) [24], Catch Fish Optimization Algorithm (CFOA) [25], Social Network Search (SNS) [26], and Football Team Training Algorithm (FTTA) [27]. Swarm-based algorithms are inspired by the behavior of groups of various types of organisms. Particle Swarm Optimization (PSO) is one of the most classical algorithms inspired by the behavior of bird flocks [28]. Colorni et al. introduced the Ant Colony Optimization (ACO) algorithm [29], inspired by the path selection behavior of ants. The Grey Wolf Optimizer (GWO) [29] is a method proposed by Mirjalili et al. inspired by the strict social hierarchy and hunting behavior of grey wolves. Xie et al. proposed Tuna Swarm Optimization (TSO) inspired by the spiral and parabolic foraging methods of tuna [30]. Abualigah et al. proposed the Reptile Search Algorithm (RSA) by crocodile hunting behavior [31]. More swarm-based algorithms are the Coati Optimization Algorithm (COA) [32], Dwarf Mongoose Optimization (DMO) [33], Spider Wasp Optimizer (SWO) [34], Remora Optimization Algorithm (ROA) [35], Genghis Khan Shark Optimizer (GKSO), [36], etc. Physics-based algorithms are proposed by mimicking physicochemical phenomena and laws. Simulated Annealing (SA) [37] is the most famous physics-based algorithm, inspired by the annealing phenomenon of molten metal in metallurgy. The Runge–Kutta algorithm is the optimization based on a mathematical principle that utilizes the Runge–Kutta’s fourth-order principle to estimate optimal solutions without the use of derivatives [38]. The Gravity Search Algorithm (GSA) [28] is proposed by being inspired by the law of gravity. Others include [39] the Weighted Mean of Vectors (INFO) [40], Flick’s Law Algorithm (FLA) [41], Light Spectrum Optimizer (LSO) [42], PID-based Search Algorithm (PSA) [43], and Kepler Optimization Algorithm (KOA) [44].

RIME is a physics-based metaheuristic algorithm proposed in 2023 by Su et al. [45]. In the development of fog ice, it derives power from natural processes. Many researchers are interested in its efficiency in solving various optimization problems and achieving exact solutions, such as image segmentation [46,47], path planning [48], feature selection [49,50], hyperparameter optimization [51], and disease diagnosis [52]. Like other optimization algorithms, RIME has some limitations and difficulties that need to be improved to achieve higher performance metrics. One of the main difficulties of RIME is the proper balancing of exploration and exploitation in the exploration and exploitation steps, respectively, and becoming stuck in local solutions while searching for the optimal solution is another key weakness of RIME. During the exploration phase, the RIME should explore the solution space in depth to avoid becoming stuck in local solutions, and, throughout the resource exploitation process, the RIME must maximize the use of effective solutions in order to efficiently reach the maximum output. However, these two phases cannot be optimally combined, as too much exploration increases the likelihood of slow convergence, while too much extraction may lead to a premature convergence to poorer solutions.

To overcome these drawbacks, this paper proposes a hybrid improved version of RIME called the Hybrid Estimation RIME Algorithm (HERIME). Two improved techniques are incorporated into the basic RIME, consisting of a synergistic fusion framework and roulette-selection-based fitness distance balanced strategy. In this paper, the HERIME algorithm is analyzed by comparing its performance on 41 CEC2017 and CEC2022 benchmark test functions against many state-of-the-art methods; thus, the effectiveness of the HERIME algorithm is measured. The experimental data are analyzed using the statistical methods of the Friedman test and Wilcoxon rank sum test. The experimental results show that the HERIME algorithm produces better results than the basic RIME algorithm and its competitors. Overall, the main contributions and innovations of the paper can be summarized as follows.

1. A synergistic fusion framework is proposed to achieve the effective incorporation of RIME and EDA, which facilitates a smoother transition between the global exploration and local exploitation phases, and thus enhances the overall collaborative search capability.
2. A roulette-selection-based fitness distance balanced strategy is applied to enhance population diversity, prevent the convergence to local optimal solutions, and strengthen the overall algorithm performance.
3. We comprehensively selected 42 benchmark test functions covering multiple dimensions and types to compare HERIME with other excellent optimization algorithms. After a series of experiments, significant results were achieved, fully verifying the effectiveness of HERIME.

The structure of this paper is organized as follows: Section 2 provides an in-depth analysis of the fundamental principles and a detailed implementation of the rime algorithm. Section 3 explores the innovative mechanisms and strategies incorporated into HERIME, clarifying their specific functions within the algorithm. Section 4 presents a comprehensive analysis of the experimental comparisons and results, with a particular focus on the application of the algorithm to the test functions. Finally, Section 5 summarizes the main findings of the paper, highlighting new improvements of the HERIME algorithm to the original method and its practical performance in testing. This section also provides an outlook on future research, offering potential application areas to further enhance the wider applicability of HERIME.

## 2. Standard RIME Algorithm

The RIME algorithm stands for the Rime Optimization Algorithm, which is a nature-inspired optimization algorithm based on physics principles. Theoretically, the formation of ice in nature has been used as the basic inspiration in developing this algorithm for modeling the process of optimization. In such an approach, any potential solution in a given optimization problem will be considered as an agent in a large search space, wherein the set of potential solutions develops the rime population. In this section, the mathematical model of the RIME algorithm will be presented. RIME simulates the evolution behaviors of soft rime and hard rime, incorporating a soft-rime search strategy and a mechanism for probing hard rime. This dual process balances exploration and exploitation, helping the algorithm thoroughly in investigating the solution space. Each phase is described in detail next.

### 2.1. Initialization of Rime Ice

In RIME, each individual position of the hippopotamus represents the value of the decision variable in space, and rime particles are individual vectors that make up the population matrix. The initialization phase of RIME is generated by randomly initializing the population like other meta-heuristic algorithms. In this step, the equation for generating the decision variable vector is shown in Equation (1).

(1) $$X_{i}=\left[x_{i,1},x_{i,2},\cdots ,x_{i,D}\right]=lb+rand\times \left(ub-lb\right),\;i=1,2,\cdots ,Np$$

where i=1,2,⋯,Np, j=1,2,…,D. $X_{i}$ represents the *i*th individual position, and lb and ub the maximum variable upper limit and minimum variable lower limit of the problem variable, respectively. Let Np represent the total number of rime particles in the search group, and D denotes the problem dimension. The population matrix is shown in Equation (2).

(2) $$X=\left[\begin{array}{c} X_{1}\\ \vdots \\ X_{N} \end{array}\right]=\left[\begin{array}{cccc} x_{1,1} & x_{1,2} & \ldots & x_{1,D}\\ x_{2,1} & x_{2,2} & \cdots & x_{2,D}\\ \vdots & \vdots & \ddots & \vdots \\ x_{N,1} & x_{N,2} & \cdots & x_{N,D} \end{array}\right]$$

### 2.2. Soft-Rime Search Strategy

In a windy environment, the growth of soft rime is highly stochastic and covers an extremely wide area but grows slowly in the same direction. Inspired by the soft rime growth, RIME proposes a soft rime search strategy to emulate the coalescence process of each rime particle. The mathematical model is shown in Equation (3).

(3) $$X_{i,j}^{new}=X_{\mathrm{best},j}+r_{1}\times \cos\left(\frac{t\times \pi }{10\times t_{\max}}\right)\times \left(1-round\left(\frac{5\times t}{t_{\max}}\right)÷5\right)\times \left(rand\times \left(ub_{i,j}-lb_{i,j}\right)+lb_{i,j}\right),\;r_{2}<E$$

where $X_{i,j}^{new}$ is the *j*th dimension of the *i*th agent and $X_{\mathrm{best},j}$ denotes the *j*th dimension of the best rime agent. t and $t_{\max}$ denote the current iteration number and the maximum iteration number, respectively. $round\left(\cdot \right)$ is a rounded integer. The variable $r_{1}$ is a stochastically generated value between −1 and 1, while $r_{2}$ is random value between 0 and 1. The variables $ub_{i,j}$ and $lb_{i,j}$ define the upper and lower bounds of the *i*th search agent in the *j*th dimension. E denotes the attachment coefficient, which affects the coalescence efficiency of rime particles, as shown in Equation (4).

(4) $$E=\sqrt{\frac{t}{t_{\max}}}$$

### 2.3. Hard-Rime Puncture Mechanism

Under strong wind conditions, hard rime is prone to crossover as it grows in the same direction. This phenomenon is called rime puncture. The puncture phenomenon inspired the RIME algorithm to propose a hard rime puncture mechanism, which allows particles to be exchanged with each other, thus improving the convergence of the algorithm and the ability to jump out of the local optimum. The mechanism is updated as shown in Equation (5).

(5) $$X_{i,j}^{new}=X_{\mathrm{best},j},\;r_{3}<F_{norm}$$

where $r_{3}$ denotes a randomly created value within the range [−1, 1], while the normalized value of the current agent fitness value is denoted as $F_{norm}$.

### 2.4. Positive Greedy Selection Mechanism

In RIME, a greedy mechanism is proposed to include the judgment of each individual in addition to judging the update of the optimal agent. Specifically, if the updated agent is better than the old agent, the old agent is replaced with the updated agent. Meanwhile, the goodness between that individual and the optimal agent is evaluated, and the better one is used as the optimal agent. The mathematical model of greedy selection mechanism is as follows:

(6) $$F\left(X_{i}\right)=F\left(X_{i}^{new}\right),\;X_{i}=X_{i}^{new},\;F\left(X_{i}^{new}\right)<F\left(X_{i}\right)$$

(7) $$F\left(X_{best}\right)=F\left(X_{i}^{new}\right),\;X_{best}=X_{i}^{new},\;F\left(X_{i}^{new}\right)<F\left(X_{best}\right)$$

The complete process of the RIME algorithm is outlined in Algorithm 1.
**Algorithm 1:** The RIME algorithm**Input**: *FEs*, *FEs*_max_, *Np*, *D*, *lb*, *ub***Output**: *X*_best_, *F*(*X*_best_)1Initialize the population randomly (*X*_1_, *X*_2_, *X*_3_, …, *X_Np_*)2Calculate *F*(*X*_i_) for all *X*_i_3Select *X*_best_, *F*(*X*_best_)4**While** (*FEs* < *FEs*_max_) **do**5      Calculate parameter *E* using Equation (4)6      **For** *i* = 1 to *Np* **do**7                 **For** *j* = 1 to *D* **do**8                       **If** *r*_1_ < *E* **then**9                             Update rime agent using Equation (3)10**                       End-If**11                       **If**
*r*_3_ < *F*_norm_ **then**12                             Update rime agent using Equation (5)13
                       **End-If**
14
                 **End-For**
15           Check boundary constraint of *X*_i_^new^16           Select the best agent and replace the suboptimal agent using Equations (6) and (7)17
      **End-For**
18      Update *X*_best_, *F*(*X*_best_)19      *FEs* = *FEs* + *Np*20**End-While**21Return *X*_best_, *F*(*X*_best_)

## 3. Proposed HERIME Algorithm

RIME guides exploration and exploitation by employing current optimal search agents. While this approach is effective in maximizing productivity and achieving the best possible results quickly on a global scale, it has difficulties when dealing with complex objective functions with high dimensionality. This limitation is evident in terms of the convergence ability and susceptibility to falling into local optima, especially in the absence of rapid solutions. Specifically, RIME performs a global search mainly around the current optimal agent during the search process, which may lead to the narrowing of the search scope and performance degradation. In addition, RIME has difficulty coordinating global and local search. Inadequate global search can cause the algorithm to fall into a local optimum, while inadequate local search can prevent the algorithm from obtaining an accurate global optimum. To address these concerns, this paper proposes two improvement strategies to boost the performance of RIME. Then, the time complexity of the HERIME algorithm proposed in this paper is analyzed, presenting pseudo-code and flowcharts. The details will be introduced in the subsequent sections.

### 3.1. Estimation of Distribution Algorithm (EDA)

As a model-based evolutionary method, the EDA can extract the descending direction of objective function fitness by fitting the distribution characteristic of the dominant population. In the EDA, the weighted maximum likelihood estimation method is used to estimate the distribution model, and the first half of the promising solutions are taken as the dominant population. The mathematical model of the EDA is described as follows.

(8) $$X_{mean}=\sum_{i=1}^{Np/2}\omega_{i}\times X_{i}$$

(9) $$\omega_{i}=\frac{\ln\left(Np/2+0.5\right)-\ln\left(i\right)}{\sum_{i=1}^{Np/2}\left(\ln\left(Np/2+0.5\right)-\ln\left(i\right)\right)}$$

(10) $$Cov=\frac{1}{0.5Np}\sum_{i=1}^{0.5Np}\left(X_{i}-X_{mean}\right)\times {\left(X_{i}-X_{mean}\right)}^{T}$$

(11) $$X_{i}=Gaussian(X_{mean},Cov)+r_{4}\times (X_{mean}-X_{i})$$

where $X_{mean}$ represents the weighted mean value of the dominant population. $\omega_{i}$ denotes the weight coefficients, arranged in descending order according to the fitness value in the dominant population. Cov is the weighted covariance matrix of the dominant population. $r_{4}\in (0,1)$ is a uniformly distributed random number. The EDA mainly chooses a high-quality solution to construct a probability distribution model; thus, it easily extracts the descending direction of the fitness value. However, the distribution model is overfitted in the later stage of iterations, resulting in a reduced population diversity and falling into local optima. Therefore, HERIME mainly employs the exploration ability of the EDA and embeds it into the RIME using synergistic fusion framework.

### 3.2. Synergistic Fusion Framework (SFF)

**Figure 2:**
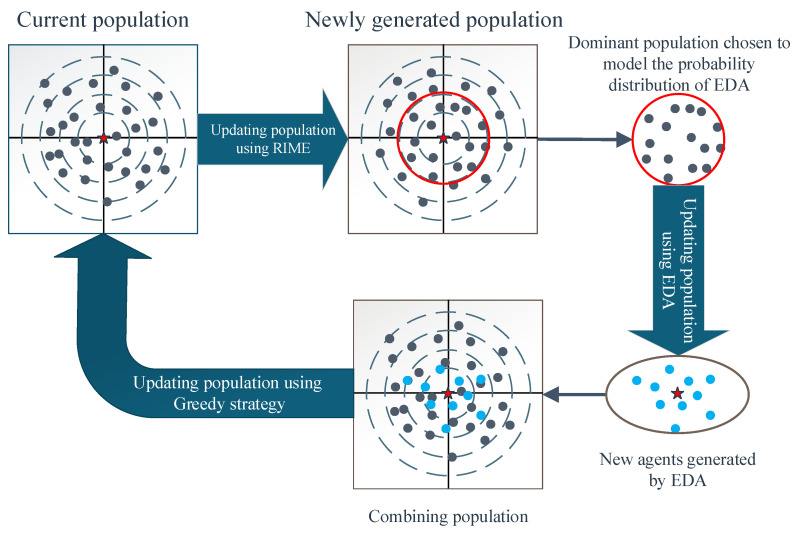
Sketch for procedure of the synergistic fusion framework.

How to effectively balance the different search behaviors of the two algorithms to improve the performance of the new algorithm is a key issue in hybrid algorithms. In order to achieve this goal, this paper develops a synergistic fusion framework. Specifically, the method establishes a link between the RIME-generated offspring population and the EDA-generated offspring population, as shown in Figure 2. In SFF, HERIME first applies the RIME algorithm to update the agents and uses a greedy selection mechanism to form the offspring population. The EDA uses maximum likelihood estimation to model the probability distributions of the agents from the top half of the fitness rankings in the offspring population, and then generates a number of agents. The RIME-generated and EDA-generated agents are retained for the next generation to participate in iteration through a greedy strategy. Since the dominant individuals chosen to build the probability distribution model are not all generated by the EDA, the overfitting flaw of the EDA can be mitigated. RIME then improves its weak global search capability by using the EDA-generated individuals to guide the RIME’s individuals to search towards more promising regions.

### 3.3. Roulette-Selection-Based Fitness Distance Balanced Strategy (RSFDB)

In the original hard fog piercing mechanism, the fog particles choose to inherit the position of the best agent. When the best agent falls into the local optimum, such an update method can hardly help RIME jump out of the local optimum. In addition, the optimal position update based on intelligence ignores other search regions, weakening the global search ability of the algorithm. For that reason, this paper proposes a roulette-selection-based fitness distance balanced strategy (RSFDB). RSFDB is an enhanced version of FDB, which incorporates the idea of roulette selection. FDB is a method designed to provide more effective guidance by calculating the scores of candidate solutions based on fitness and distance values and selecting candidate solutions from the population based on the modified scores [53]. RIME inherits the optimal agent’s hard freezing puncture mechanism, ignoring the characteristics of the other dominant individuals. Such a selection severely diminishes the population diversity and leads to the algorithm’s susceptibility to fall into the local optimality trap. FDB selects only the agent with the highest scores at each iteration, and RSFDB will choose an agent randomly from the population according to the probability based on the roulette selection mechanism. Note that the higher the score, the higher the probability that an agent will be selected. This ensures both sufficiently robust development capabilities and decent exploration capabilities.

The procedure for the RSFDB strategy is shown below:

Step 1: Calculate the Euclidean distance between two agents $X_{i}$ and the optimal agent $X_{\mathrm{best}}$ with the following formula:

(12) $$Dis_{i}=\sqrt{{\left(X_{i,1}-X_{\mathrm{best},1}\right)}^{2}+{\left(X_{i,2}-X_{\mathrm{best},2}\right)}^{2}+\ldots +{\left(X_{i,D}-X_{\mathrm{best},D}\right)}^{2}}$$

Step 2: Create the distance vector $Dis_{X}$ using the Euclidean distance values for each individual.

(13) $$Dis_{X}=\left[Dis_{1};Dis_{2};\ldots ;Dis_{Np}\right]$$

Step 3: Calculate the score for each feasible solution using the fitness value and the distance vector by Equation (14).

(14) $$Sco_{i}=\left(1-\lambda \right)\times norm\left(F\left(X_{i}\right)\right)+\lambda \times norm\left(Dis_{i}\right)$$

Step 4: Create the score vector $Sco_{X}$.

(15) $$Sco_{X}=\left[Sco_{1};Sco_{2};\ldots ;Sco_{Np}\right]$$

Step 5: Select the guided solution for each individual according to $Sco_{X}$ using a roulette wheel approach.

(16) $$X_{RSFDB}=select(Sco_{X})$$

Step 6: Replace $X_{\mathrm{best}}$ with $X_{RSFDB}$ in Equation (5); the formula is as follows:

(17) $$X_{i,j}^{new}=X_{RSFDB,j},\;r_{3}<F_{norm}$$

Enriching the diversity of the RIME population and ameliorating the imbalance between exploration and exploitation by providing each agent with a reference agent that has a high probability of being a dominant solution through the RSFDB.

**Figure 3:**
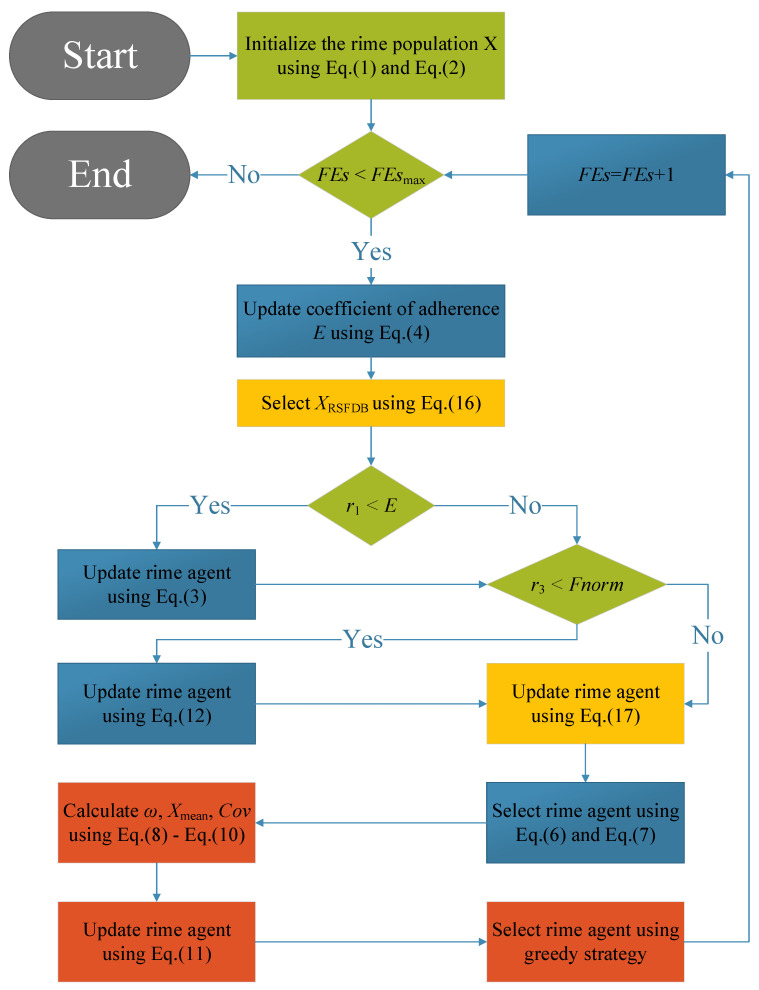
The flowchart of proposed HERIME algorithm.

The main steps of the proposed HERIME are depicted in Algorithm 2 and visualized for more details in Figure 3.
**Algorithm 2:** The HERIME algorithm**Input**: *FEs*, *FEs*_max_, *Np*, *D*, *lb*, *ub*, *λ***Output**: *X*_best_, *F*(*X*_best_)1Initialize the population randomly (*X*_1_, *X*_2_, *X*_3_, …, *X_Np_*)2Calculate *F*(*X*_i_) for all *X*_i_3Select *X*_best_, *F*(*X*_best_)4**While** (*FEs* < *FEs*_max_) **do**5      Calculate parameter *E* using Equation (4)6      Select *X*_RSFDB_ using Equation (16)7      **For** *i* = 1 to *Np* **do**8                 **For** *j* = 1 to *D* **do**9                       **If** *r*_1_ < *E* **then**10                             Update rime agent using Equation (3)11
                       **End-If**
12                       **If**
*r*_3_ < *F*_norm_ **then**13                             Update rime agent using Equation (17)14
                       **End-If**
15
                 **End-For**
16          Check boundary constraint of *X*_i_^new^17          Select the best agent and replace the suboptimal agent using Equations (6) and (7)18
      **End-For**
19      *FEs* = *FEs* + *Np*20      Calculate ω, *X*_mean_, *Cov* using Equations (8)–(10)21      Generate *Np*_e_ agents using Equation (11)22      Update population using greedy strategy23      *FEs* = *FEs* + *Np*_e_24      Update *X*_best_, *F*(*X*_best_)25**End-While**26Return *X*_best_, *F*(*X*_best_)

### 3.4. Time Complexity

The algorithm’s time complexity is determined by the variables dimension *D*, population size *Np*, and number of iterations *T*. The temporal complexity of the original RIME algorithm is dictated by the initialization and update operators, namely, the soft rime and hard rime. It can be mathematically represented as follows:

$$O\left(RIME\right)=O\left(initialization\right)+O\left(RIME\;operators\right)=O\left(Np\times D\right)+O\left(Np\times D\times T\right)≅O\left(Np\times D\right)$$

The proposed HERIME differs from the RIME in the addition of a synergistic fusion framework (SFF) and roulette-selection-based fitness distance balanced strategy (RSFDB) in each iteration. RSFDB is used to generate reference agents to replace the optimal individuals of Equation (5), so there is no impact on the time complexity. SFF newly generates *Np*_e_ agents at each iteration, so its time complexity is $O\left(Np_{e}\times D\times T\right)$. To sum up, the time complexity of HERIME is as follows:

$$\begin{array}{l} O\left(HERIME\right)=O\left(initialization\right)+O\left(RIME\;operators\right)+O\left(SFF\right)\\ \;\;\;\;\;\;\;\;\;\;\;\;\;\;\;\;\;\;\;\;\;\;\;=O\left(Np\times D\right)+O\left(Np\times D\times T\right)+O\left(Np_{e}\times D\times T\right)\\ \;\;\;\;\;\;\;\;\;\;\;\;\;\;\;\;\;\;\;\;\;\;\;≅O\left(\left(Np+Np_{e}\right)\times D\times T\right) \end{array}$$

Although the time complexity of HERIME has increased compared to RIME, this paper uses the maximum number of evaluations as the termination condition, which allows a fair comparison of the performance between the two algorithms. The final experimental results also confirm that the performance of HERIME is superior to the basic RIME.

## 4. Experimental Results and Analysis

This section first examines the effectiveness of two improvement strategies integrated into HERIME. Then, the performance of HERIME is analyzed and compared with several classical advanced algorithms together with some high-performance improved algorithms.

### 4.1. Experimental Setup and Evaluation Criteria

All experiments were conducted on a CPU with properties of AMD R9 7950X @ 5.20 GHz, 64 GB RAM, running on the Windows 10 operating system. All code was implemented in MATLAB R2021b.

This study employs the widely used and authoritative CEC2017 benchmark suite and the CEC2022 benchmark suite. The benchmark suite test functions are divided into four types: unimodal functions with a single global optimum, to evaluate the accuracy and convergence speed in simple optimization problems; multimodal functions with multiple local optima, to assess the ability to find the global optimal solution and the fine local search capability of the algorithm; and hybrid and composite functions, designed to measure the algorithm’s ability to handle complex continuous problems. The specific details of the CEC2017 benchmark suite are given in Table 1. The specific details of the CEC2022 benchmark suite are provided in Table 2.

In this paper, HERIME is compared with nine metaheuristic algorithms including the basic RIME. These algorithms include not only the basic algorithms that have become more popular in recent years, EO, SAO, and MRFO, but also five advanced improved algorithms, IYDSE, DFDBARO, EOSMA, IGWO, and LSHADE. Table 3 lists the parameter settings for each algorithm. To reduce the effect of randomness on the experimental results, we executed 51 identical trials for each optimization algorithm in all tests. Ensuring the fairness of the algorithmic experiments requires that each algorithm performs the same number of function evaluations on the test functions. From an Oracle-based computational point of view [15], this ensures that comparisons between algorithms are consistent. Therefore, for a fair evaluation, we set the maximum number of evaluations for each algorithm to 1000 × D. Due to the varying difficulty of solving testing problems in different dimensions (D), in order to ensure the balance of the experiment, two different dimensions were selected to test the optimization algorithm in the testing sets CEC2017 and CEC2022, namely, 10D, 30D, 50D, and 100D, and 10D and 20D, respectively. This can make the experimental results more convincing. All experiments were conducted with these parameter settings.

The optimal value (Best), mean value (Ave), and standard deviation (Std) are used as criterions of the algorithms. The Friedman test and Wilcoxon rank sum test are used to analyze the experimental results. All comparison algorithms are ranked using the Friedman test, with higher rankings indicating better performance. The Wilcoxon signed-rank test is set to a 5% significance level, with the symbols “(+/=/−)” indicating that HERIME performs better, similar to, or worse than its competitors, respectively.

### 4.2. Ablation Study on the Fusion Strategy for HERIME

As shown in Section 3, HERIME integrates the synergistic fusion framework and roulette-selection-based fitness distance balanced strategy into the basic RIME. To evaluate the impact of each improvement technique, we compared RIME with CRIME, RRIME, and HERIME on two test suites. Table 4 describes these two variants. In Table 4, the value “YES” indicates that the corresponding strategy is included in RIME, while the value “NO” indicates no integration. For example, CRIME indicates that RIME applies only the synergistic fusion framework. The results of the Friedman test and Wilcoxon rank sum test for these algorithms are summarized in Table 5 and Table 6, respectively.

**Figure 4:**
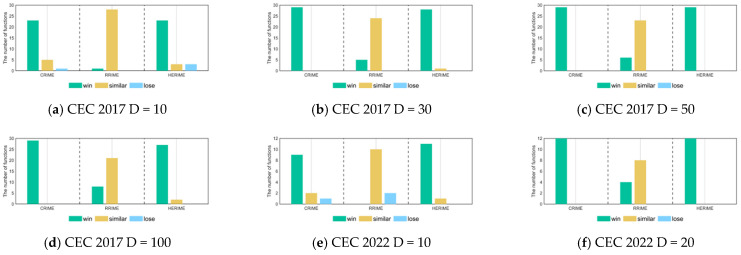
The visualization of Wilcoxon rank sum test results for RIME, CRIME, RRIME, and HERIME based on CEC 2017 and CEC 2022.

Table 5 shows the results of the Friedman test for the different cases, showing the average rankings of the test sets for the different dimensions as well as the overall average rankings of the set of test functions. We can learn that there are significant differences between the three RIME variants and the basic RIME. And HERIME, which integrates all the optimization techniques, achieved the smallest average value of 1.27, which indicates that it has the best performance among all the algorithms. In contrast, RIME ranked last with an overall mean ranking of 3.61. CRIME and RRIME had overall mean rankings of 1.86 and 3.25, respectively. These results further illustrate the effectiveness of the improved strategies, showing that integrating them significantly improves the algorithm performance. Figure 4 visualizes the results of the Wilcoxon rank sum test between the RIME and the three variants based on Table 6. We can clearly notice that CRIME has individual functions inferior (similar) to the basic RIME only when dealing with low-dimensionality problems. Although the performance of RRIME is similar to that of RIME for most functions, there is no degradation in its performance after integrating the RSFDB technique. In the CEC2017 10D/30D/50D/100D test functions, HERIME demonstrates better performance than basic RIME in 23/28/29/27 functions and is inferior to RIME in three functions only on 10D. Similarly, HERIME demonstrates better performance than basic RIME in 11/12 functions in CEC2022 10D/20D, with RIME showing comparable performance on 1 function.

The ablation experiments show that the integration of multiple strategies significantly improves the performance and enhances the stability and local search capability of HERIME.

### 4.3. Results Analysis for HERIME and Competitors

**Figure 5:**
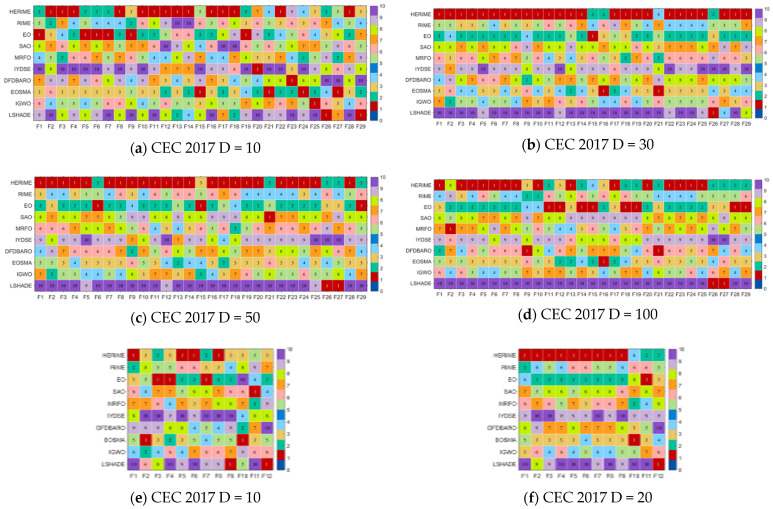
The ranking heatmap of HERIME and competitors in solving CEC2017/CEC2022.

**Figure 6:**
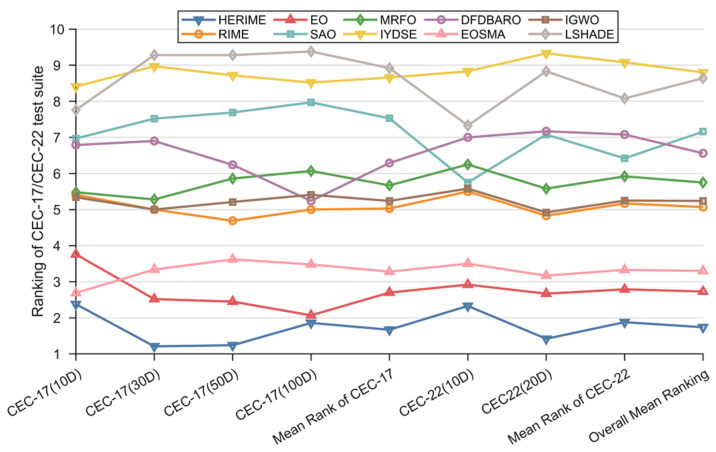
The visualization of Friedman test results of HERIME and competitors in solving CEC2017/CEC2022.

This subsection evaluates the performance of HERIME in global optimization problems using the 10/30/50/100-dimension CEC2017 test suite and the 10/20-dimension CEC2022 test suite. The results of the experiments containing Best/Ave/Std/Rank are shown in Appendix A, Table A1, Table A2, Table A3, Table A4, Table A5 and Table A6. As a visual measure of the performance of HERIME and the competing algorithms on different functions, Figure 5 shows a ranked heatmap based on the mean values. Table 7 presents the Friedman mean rankings of each algorithm on CEC2017 for 10, 30, 50, and 100 dimensions and CEC2022 for 10 and 20 dimensions, visualized in Figure 6.

**Figure 7:**
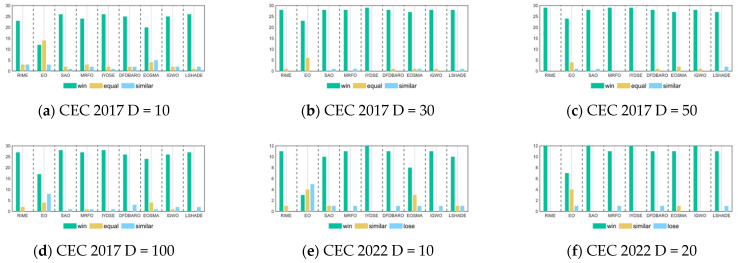
The visualization of Wilcoxon rank sum test results of HERIME and competitors in solving CEC2017/CEC2022.

The Wilcoxon rank sum test was conducted to fully evaluate the performance of HERIME algorithms, comparing HERIME with RIME, EO, SAO, MRFO, IYDSE, DFDBARO, EOSMA, IGWO, and LSHADE. A nonparametric statistical method, the Wilcoxon rank sum test, was employed with a significance level of α = 0.05, based on 51 independent executions of each algorithm. The null hypothesis asserts that there is no statistically significant difference between the solutions obtained by HERIME and the comparison algorithms on the same benchmark function. The “+” symbol indicates the rejection of the null hypothesis, indicating that HERIME significantly outperforms the comparison algorithm at the 95% confidence level. On the contrary, the symbol “−” also indicates the rejection of the null hypothesis, but, in this case, it means that HERIME underperforms relative to the comparison algorithm. The “=” symbol indicates that the null hypothesis is not rejected, which means that there is no significant difference in performance between HERIME and the other algorithms. Table 8 summarizes the total number of benchmark functions belonging to the “+”, “=”, and “−” categories for HERIME and the comparison algorithms, respectively, as visualized in Figure 7. The detailed summary of the findings is as follows:

(1) CEC2017 10-Dimensional results: HERIME achieved the best results on 14 out of all 29 functions and ranked second on 6 functions. In the Friedman test, HERIME ranked first with an average score of 2.38. RIME ranked fifth with an average score of 5.41. The Friedman *p*-value of Table 7 is less than 0.05, indicating that there is a difference in the performance of these algorithms in solving CEC2017 10D. In the Wilcoxon rank sum test, the number of “+” symbols is much higher than the number of “−” symbols, meaning that HERIME significantly outperforms the other algorithms in most of the functions, as follows: HERIME is superior (inferior) to RIME, EO, SAO, MRFO, IYDSE, DFDBARO, EOSMA, IGWO, and LSHADE on the 23(3), 12(3), 26(1), 24(2), 26(1), 25(2), 20(5), 25(2), and 26(2) test functions. That is, HERIME beats RIME, EO, SAO, MRFO, IYDSE, DFDBARO, EOSMA, IGWO, and LSHADE.

(2) CEC 2017 30-Dimensional results: HERIME achieved the best scores on 25 of all 29 functions and ranked second on 3 functions. In the Friedman test, HERIME ranks first with an average score of 1.21. RIME ranks fifth with an average score of 5.00. The Friedman *p*-value in Table 7 is less than 0.05, indicating that there is a difference in the performance of these algorithms in solving CEC2017 30D. In the Wilcoxon rank sum test, the number of symbols “+” is much higher than the number of symbols “−”, which means that HERIME significantly outperforms the other algorithms in most functions. Remarkably, the total number of “−” symbols obtained by HERIME on CEC 2017 30D functions is only four, and HERIME is not weaker than RIME, EO, IYDSE, DFDBARO, and IGWO. As shown below, HERIME outperforms (underperforms) RIME, EO, SAO, MRFO, IYDSE, DFDBARO, EOSMA, IGWO, and LSHADE on the 28(0), 23(0), 28(1), 28(0), 29(0), 28(0), 27(1), 28(0), and 28(1) test functions. The HERIME algorithm maintains excellent optimization performance as the dimensionality and complexity increases.

(3) CEC2017 50-dimensional results: HERIME achieved the best scores on 23 out of all 29 functions and ranked second on 5 functions. In the Friedman test, HERIME ranks first with an average score of 1.24. RIME ranks fourth with an average score of 4.69. The Friedman *p*-value in Table 7 is less than 0.05, indicating that there is a difference in the performance of these algorithms in solving CEC2017 50D. In the Wilcoxon rank sum test, the number of symbols “+” is much higher than the number of symbols “−”, which means that HERIME significantly outperforms the other algorithms in most functions. It is worth noting that the total number of “−” symbols obtained by HERIME on CEC 2017 50D functions is only four. For RIME, MRFO, IYDSE, EOSMA, DFDBARO, and IGWO, HERIME is not weaker than them. This is shown as follows: HERIME outperforms (underperforms) RIME, EO, SAO, MRFO, IYDSE, DFDBARO, EOSMA, IGWO, and LSHADE on the 29(0), 24(1), 28(0), 29(0), 29(0), 28(0), 27(0), 28(0), and 27(2) test functions. In conclusion, the HERIME algorithm gives satisfactory solutions consistently as the dimensionality and complexity increase.

(4) CEC2017 100-dimensional results: HERIME achieved the best scores on 15 out of all 29 functions and ranked second on 9 functions. In the Friedman test, HERIME ranks first with an average score of 1.86. RIME ranks fourth with an average score of 5.00. The Friedman *p*-value in Table 7 is less than 0.05, indicating that there is a difference in the performance of these algorithms in solving CEC2017 50D. In the Wilcoxon rank sum test, the number of symbols “+” is much higher than the number of symbols “−”, which means that HERIME significantly outperforms the other algorithms in most functions. It is worth noting that HERIME is not weaker than RIME. This is shown below: HERIME outperforms (underperforms) RIME, EO, SAO, MRFO, IYDSE, DFDBARO, EOSMA, IGWO, and LSHADE for the 27(2), 17(8), 28(1), 27(1), 28(1), 26(3), 24(1), 26(2), and 27(2) test functions. Although HERIME does not perform as well in CEC2017 100D as it does in CEC2017 50D, it is still the best optimization method in terms of overall performance.

(5) CEC2022 10D results: HERIME achieves the best results on 4 out of all 12 functions, ranks second on 2 functions, and third on 5 functions. HERIME ranks fifth on F11 but still outperforms the basic RIME. On the Friedman test, HERIME ranks first with a mean score of 2.33. RIME ranks fourth with a mean score of 5.50. The Friedman *p*-value in Table 7 is less than 0.05, indicating that there is a difference in the performance of these algorithms in solving CEC2022 10D. In the Wilcoxon rank sum test, HERIME obtains a much higher number of “+” signs than “−” signs, which means that HERIME significantly outperforms other algorithms except EO in most functions. This means that HERIME is significantly better than other algorithms other than EO in most functions. It is worth noting that HERIME is not weaker than RIME. This is shown below: HERIME outperforms (underperforms) RIME, EO, SAO, MRFO, IYDSE, DFDBARO, EOSMA, IGWO, and LSHADE for the 11(0), 3(5), 10(1), 11(1), 12(0), 11(1), 8(1), 11(1), and 10(1) test functions. Although HERIME is not as good as EO on the Wilcoxon rank sum test, it ranks better than EO on the Friedman test. This phenomenon occurs because the difference between the two algorithms is not significant on some of the functions, but there is still a difference. Therefore, we can still conclude that HERIME has the best overall performance on the CEC2022 10D test functions.

(6) CEC2022 20D results: HERIME achieves the best results on 9 out of all 12 functions and ranks second on 2. HERIME ranks fourth on F10 but still outperforms the basic RIME. On the Friedman test, HERIME ranks first with an average score of 1.42. RIME ranks fourth with an average score of 4.83 (Table 7). The Friedman *p*-values are less than 0.05, indicating that there is a difference in the performance of these algorithms in solving CEC2022 10D. In the Wilcoxon rank sum test, HERIME obtains a much higher number of “+” signs than “−” signs, which implies that HERIME significantly outperforms other algorithms than EO in most functions. It is worth noting that HERIME is not weaker than RIME, SAO, IYDSE, EOSMA, and IGWO. This is shown below: HERIME outperforms (underperforms) RIME, EO, SAO, MRFO, IYDSE, DFDBARO, EOSMA, IGWO, and LSHADE for the 12(0), 7(1), 12(0), 11(1), 12(0), 11(1), 11(0), 12(0), and 11(1) test functions. Compared to the results of CEC2022 10D, HERIME maintains a better performance after the dimensionality rise and, therefore, achieves better overall results. HERIME is superior on both statistical tests compared to EO. In conclusion, HERIME has robust capabilities in this test set.

**Figure 8:**
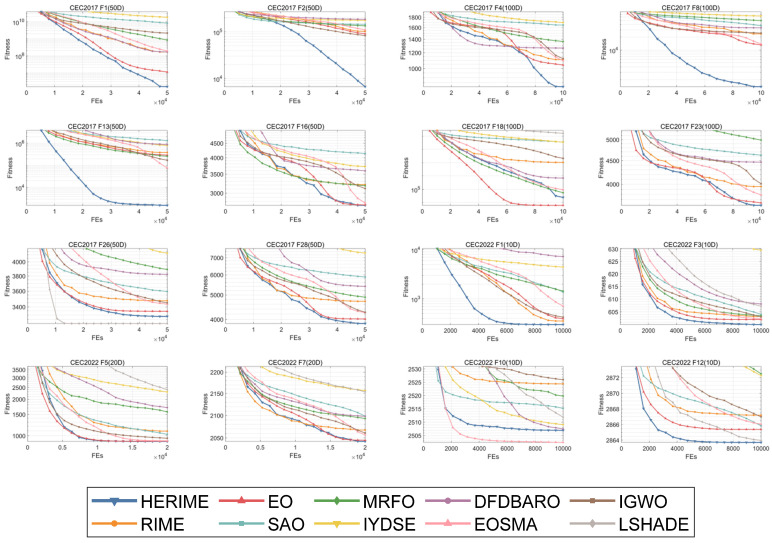
The convergence curves of HERIME and competitors in solving CEC2017/CEC2022.

**Figure 9:**
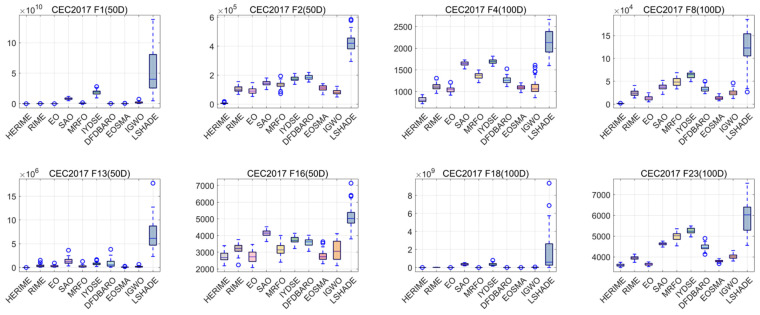

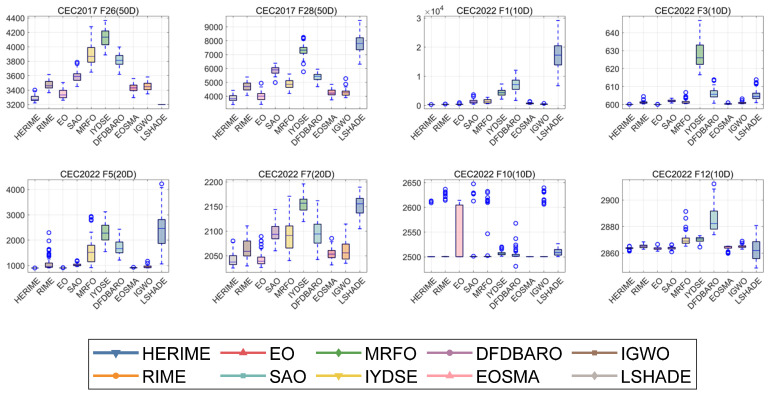
The boxplots of HERIME and competitors in solving CEC2017/CEC2022.

**Figure A1:**
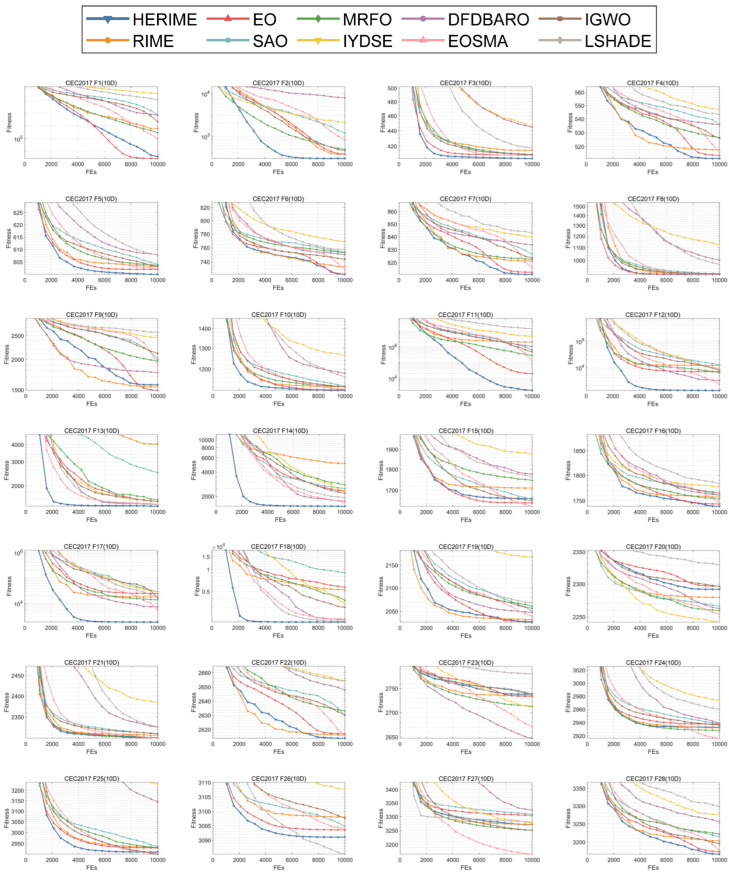

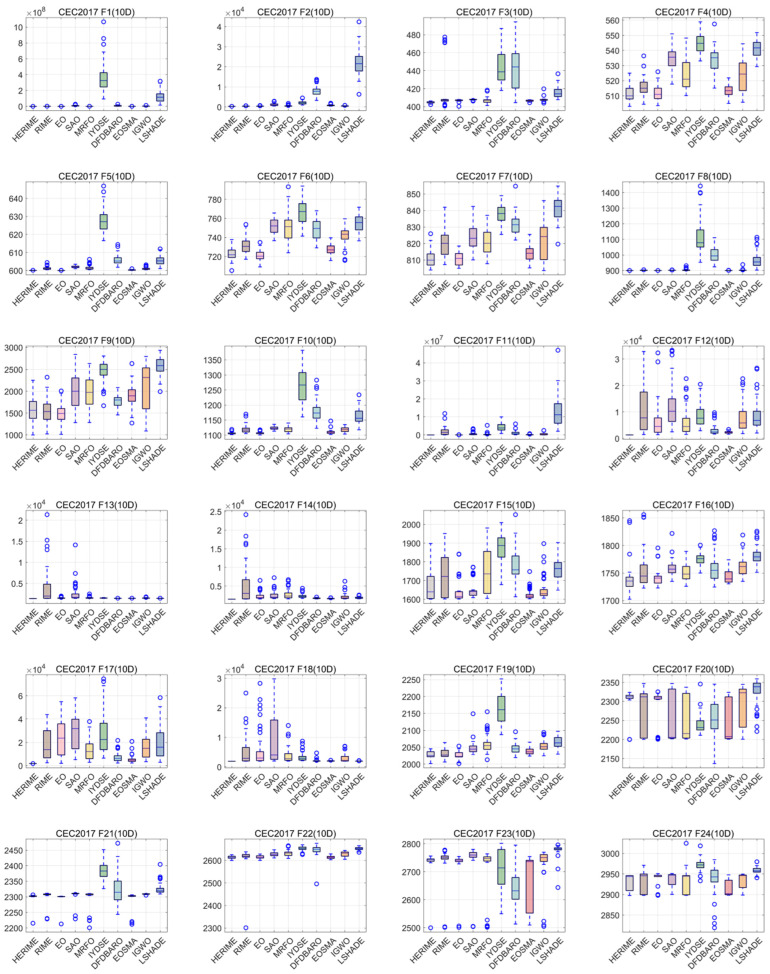

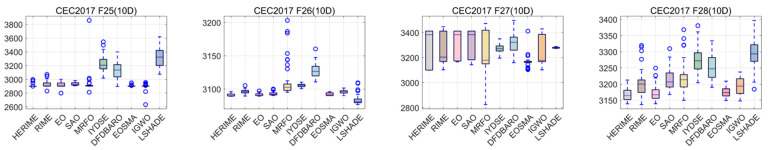
The convergence curves and boxplots of HERIME and other competitors based on CEC2017 (10D).

**Figure A2:**
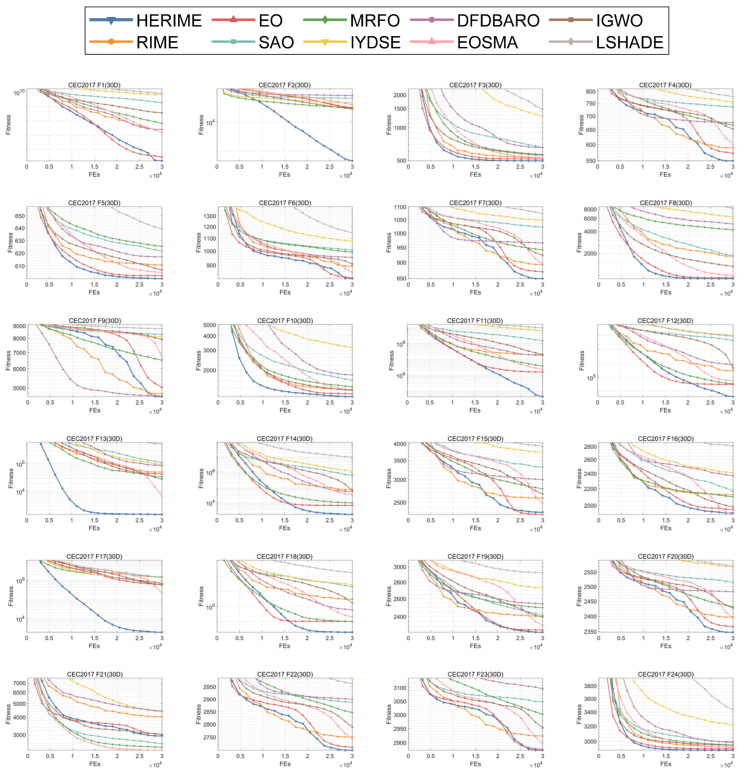

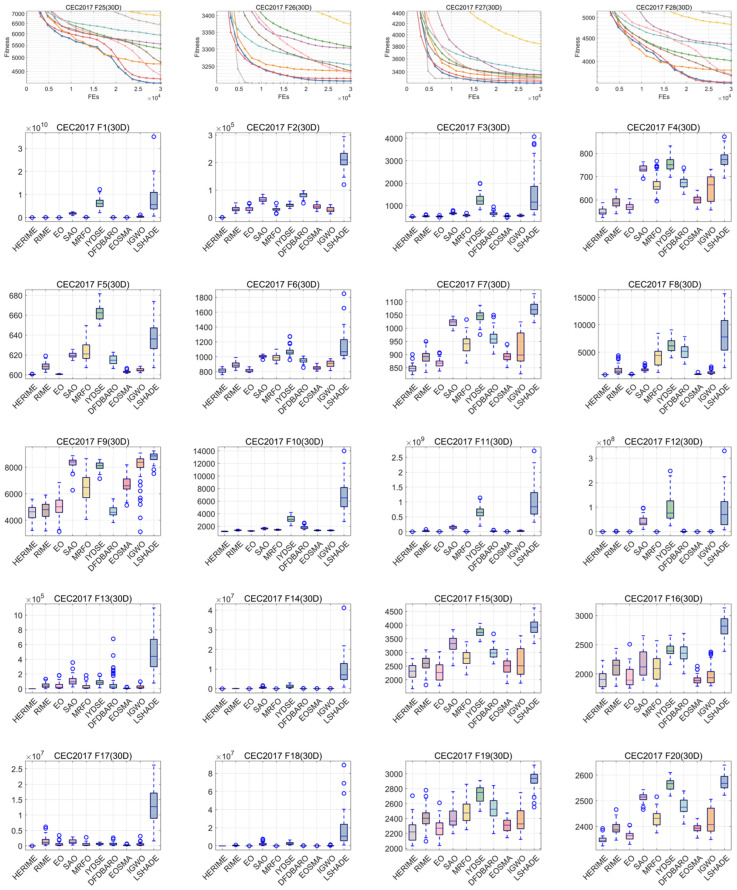

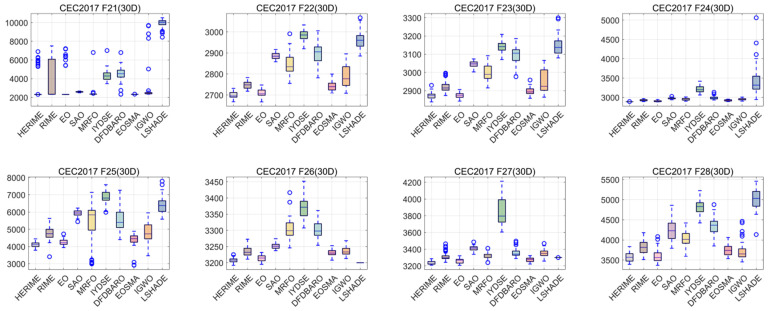
The convergence curves and boxplots of HERIME and other competitors based on CEC2017 (30D).

**Figure A3:**
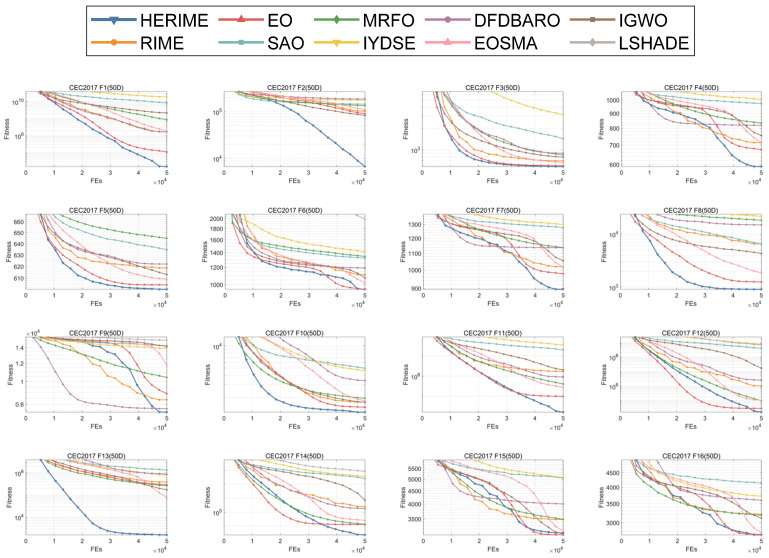

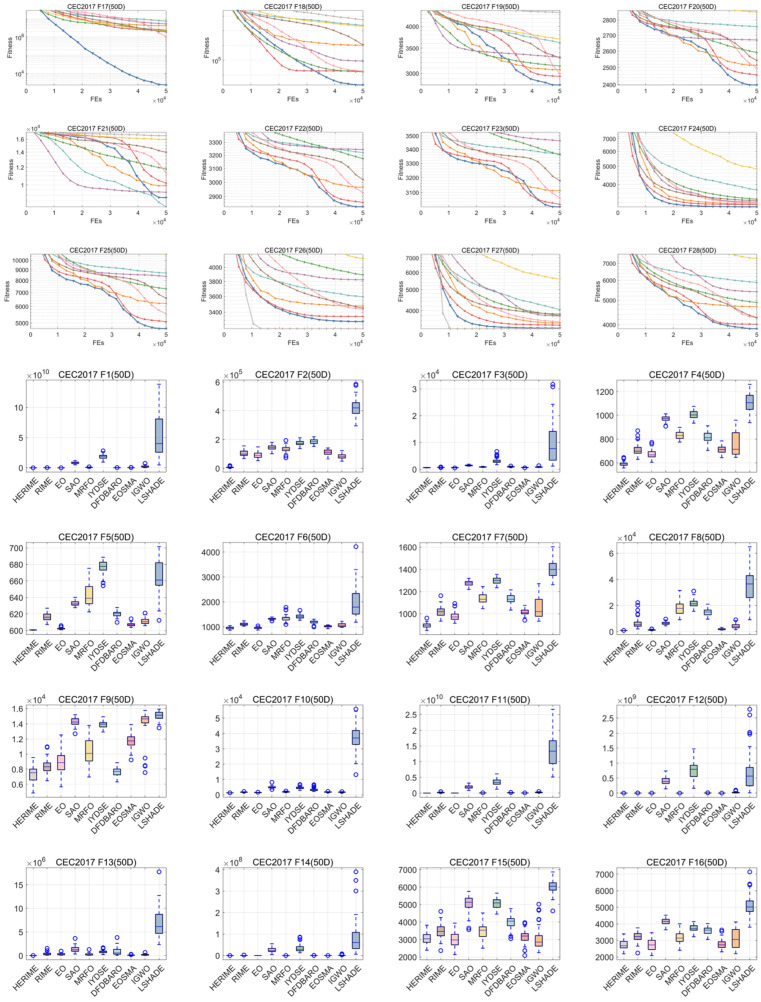

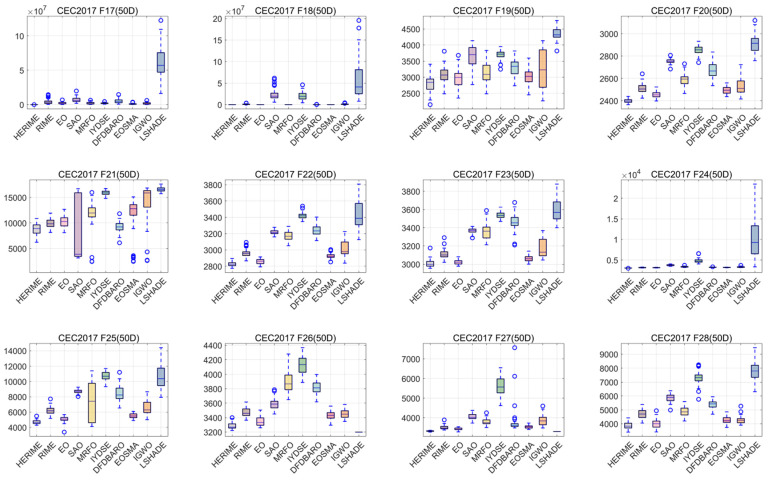
The convergence curves and boxplots of HERIME and other competitors based on CEC2017 (50D).

**Figure A4:**
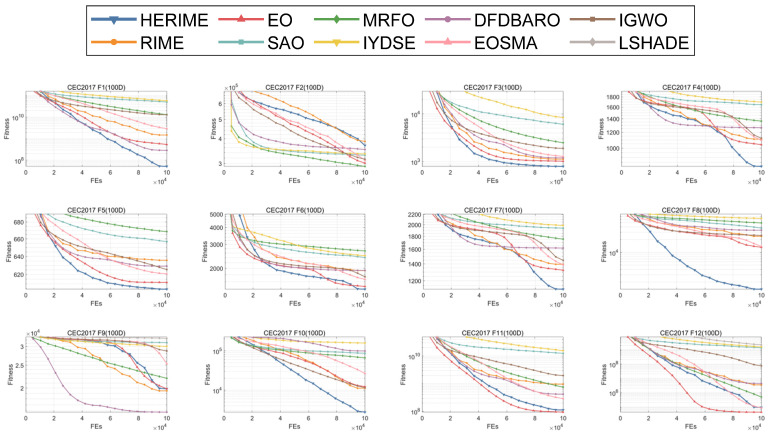

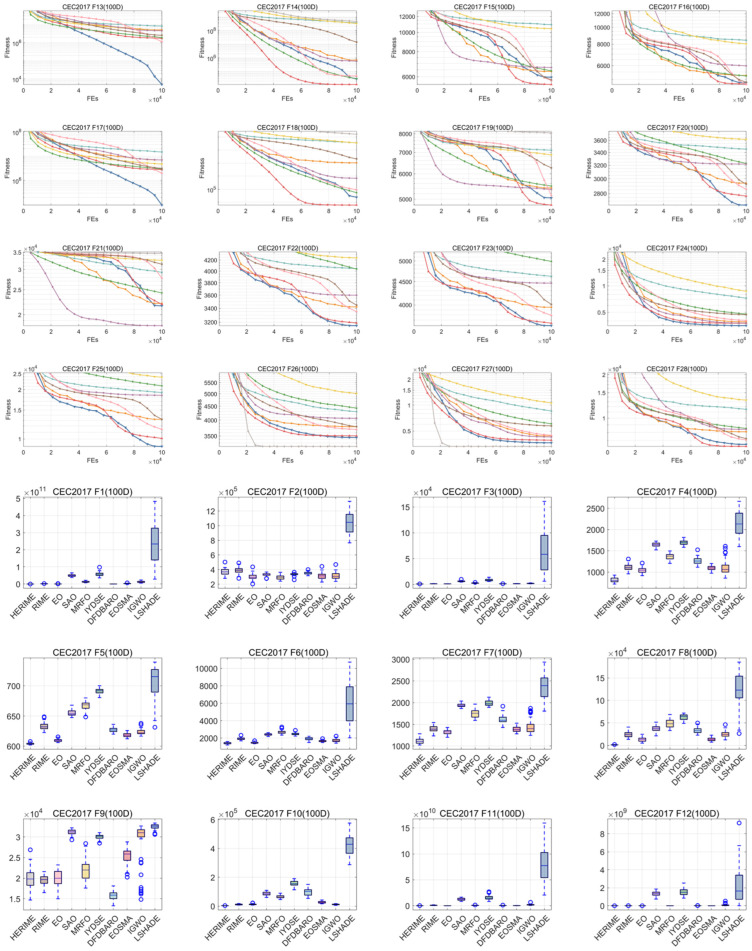

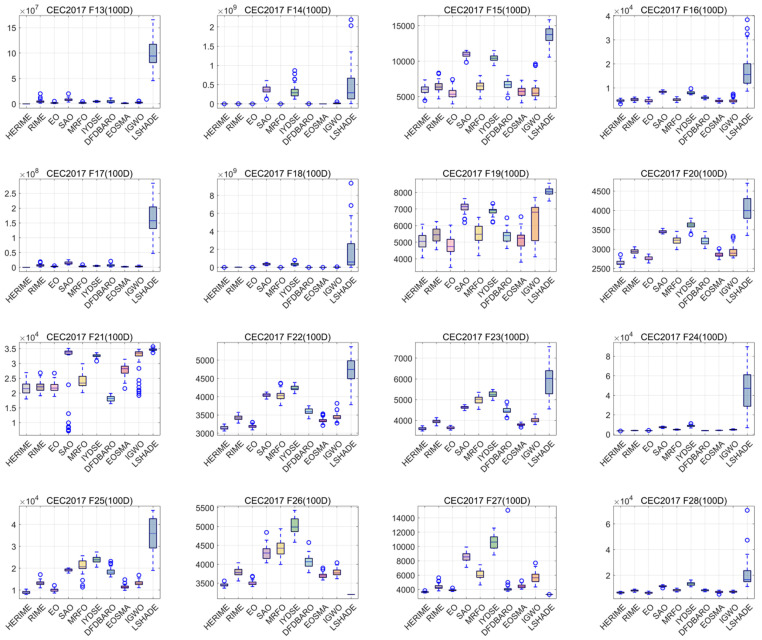
The convergence curves and boxplots of HERIME and other competitors based on CEC2017 (100D).

**Figure A5:**
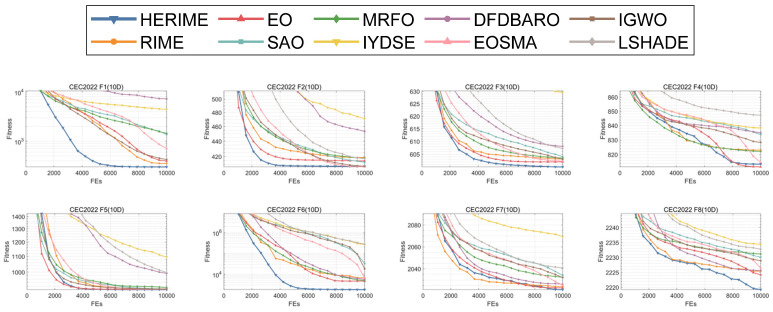

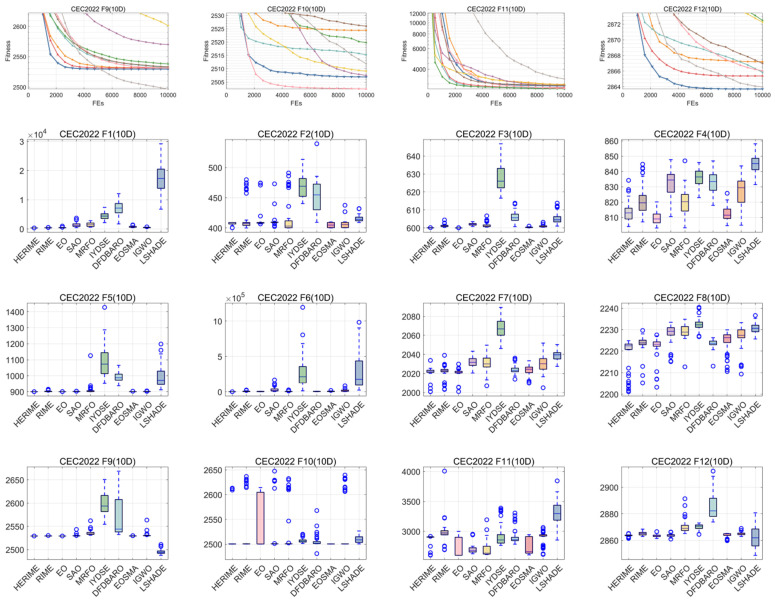
The convergence curves and boxplots of HERIME and other competitors based on CEC2022 (10D).

**Figure A6:**
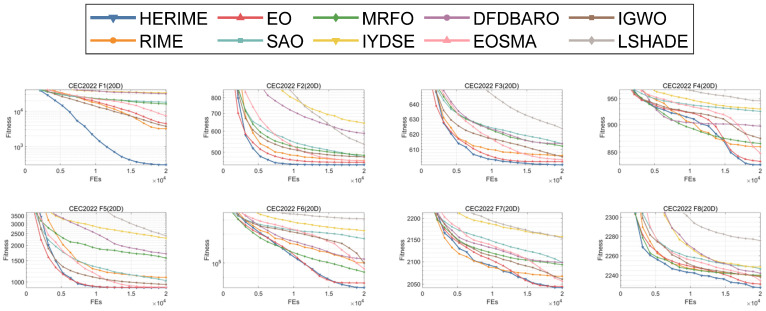

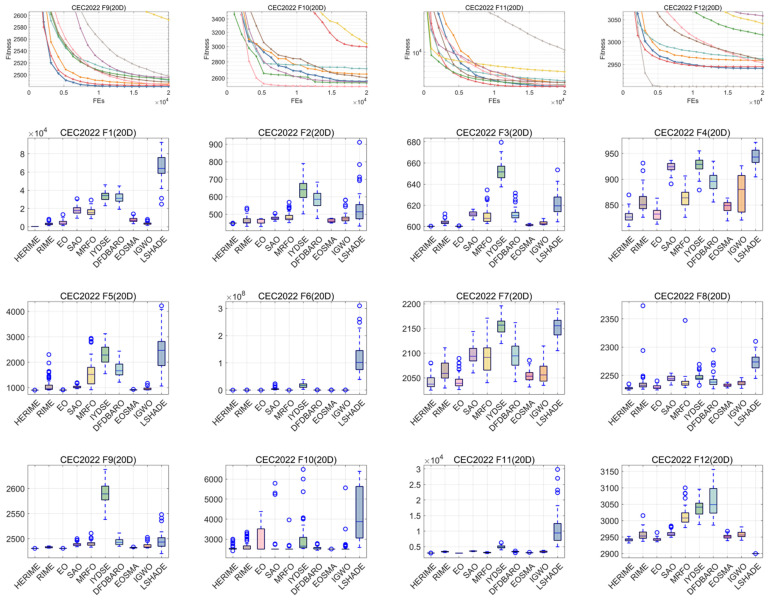
The convergence curves and boxplots of HERIME and other competitors based on CEC2022 (20D).

Furthermore, in order to visually compare the convergence trends and verify the stability of HERIME, Figure 8 and Figure 9 show the convergence graphs and boxplots of some functions under these dimensions, respectively. The whole convergence and boxplots can be obtained in Figure A1, Figure A2, Figure A3, Figure A4, Figure A5 and Figure A6 in Appendix B.

According to Figure 8, HERIME achieves better solutions with a faster convergence on most functions. For F4, F16, and F23 of CEC2017, HERIME converges slightly slower in the early stages, but is able to jump out of the local optimum in the middle and late stages to achieve the highest convergence accuracy. Note that HERIME shows better performance on both low- and high-dimensional upper functions. These results show that HERIME is good at balancing exploration and exploitation to prevent a premature convergence to a local optimum. This balance is critical because it demonstrates HERIME’s ability to maintain a comprehensive search strategy while effectively utilizing promising regions of the solution space. This good convergence performance is attributed to the synergistic fusion framework, which significantly improves the speed and accuracy of the algorithm, resulting in faster access to the optimal solution. The roulette-selection-based fitness distance balanced strategy extends the search range that makes it more capable of jumping out of the local optimum, thus improving the overall optimization performance. For F18 and F26 of CEC2017 and F10 of CEC2022, HERIME is not the best performer, but HERIME has a better convergence speed and convergence accuracy compared to the basic RIME. The overall performance of HERIME on low/high-dimensional benchmark functions highlights its advantages over the basic RIME algorithm. The results show that HERIME is a competitive optimization tool with significant improvements in both search accuracy and convergence speed.

During the validation process, we use box-and-line plots as a visualization tool to comprehensively show the performance of different algorithms on each test set. This method helps to visually compare the performance difference between HERIME and other algorithms, especially in terms of algorithm stability. The boxplot in Figure 9 shows the distribution of the test results, with the width of the box indicating the data range, the line inside the box indicating the median value, and the “o” indicating the outliers. Compared to other algorithms, the HERIME box is lower and more compact, with fewer outliers, indicating that HERIME gives a better quality and stability of solutions regarding the CEC2017/CEC2022 benchmark functions.

Based on the results of 42 benchmark functions from the CEC2017 and CEC2022 test sets, HERIME shows superior performance in different dimensions, significantly outperforming the basic RIME. The superior performance of HERIME in unimodal functions stems from the EDA methodology introduced by SFF. The EDA method successfully guides the population towards promising regions to explore, which improves the convergence accuracy and convergence speed. In addition, SFF enriches the population diversity by integrating the populations generated by the two methods, which, in turn, improves the performance of HERIME on multimodal and complex functions. RSFDB employs the roulette wheel concept, which effectively balances exploration and exploitation, while the high probability selectivity of the dominant solution ensures the convergence ability of HERIME.

## 5. Conclusions

In this paper, we propose an improved hybrid version of RIME called HERIME. HERIME uses a synergistic fusion framework to integrate EDA and incorporates a roulette-selection-based fitness distance balanced in the hard fogging phase strategy. These improved techniques are effective in avoiding local optima and providing a smooth transition between the exploration and exploitation phases. In order to validate the effectiveness of HERIME, it is evaluated on the benchmark test suite CEC2017 and CEC2022 by comparing it with eight excellent algorithms. Moreover, various visualizations of convergence curves and boxplots are provided. The experimental results show that HERIME exhibits strong performance in terms of convergence and stability, outperforming other comparison algorithms. It does not degrade the optimization performance due to dimensionality changes and exhibits a strong stability in different dimensions without significant fluctuations. Although the proposed method shows strong performance in low-dimensional and high-dimensional global optimization problems, there is still potential for further improvement. First, the integration of EDA leads to a longer execution time, and the structure of HERIME can be further optimized to reduce the computational cost. Second, integration with other AI techniques, such as reinforcement learning and deep learning, can be considered. Third, binary- and multi-objective versions of HERIME can be further investigated to provide better solutions for solving complex real-world problems. Finally, the application of the HERIME algorithm to problems such as shop floor scheduling, UAV delivery route optimization, and image segmentation will be discussed in future research.

## Figures and Tables

**Table 1:** CEC 2017 test functions.

Type	ID	CEC 2017 Function Name	Search Range	f_min_
Unimodal	F1	Shifted and Rotated Bent Cigar Function	[−100, 100]	100
	F2	Shifted and Rotated Zakharov Function	[−100, 100]	300
Multimodal	F3	Shifted and Rotated Rosenbrock’s Function	[−100, 100]	400
	F4	Shifted and Rotated Rastrigin’s Function	[−100, 100]	500
	F5	Shifted and Rotated Expanded Scaffer’s F6 Function	[−100, 100]	600
	F6	Shifted and Rotated Lunacek Bi_Rastrigin Function	[−100, 100]	700
	F7	Shifted and Rotated Non-Continuous Rastrigin’s Function	[−100, 100]	800
	F8	Shifted and Rotated Levy Function	[−100, 100]	900
	F9	Shifted and Rotated Schwefel’s Function	[−100, 100]	1000
Hybrid	F10	Hybrid Function 1 (N = 3)	[−100, 100]	1100
	F11	Hybrid Function 2 (N = 3)	[−100, 100]	1200
	F12	Hybrid Function 3 (N = 3)	[−100, 100]	1300
	F13	Hybrid Function 4 (N = 4)	[−100, 100]	1400
	F14	Hybrid Function 5 (N = 4)	[−100, 100]	1500
	F15	Hybrid Function 6 (N = 4)	[−100, 100]	1600
	F16	Hybrid Function 6 (N = 5)	[−100, 100]	1700
	F17	Hybrid Function 6 (N = 5)	[−100, 100]	1800
	F18	Hybrid Function 6 (N = 5)	[−100, 100]	1900
	F19	Hybrid Function 6 (N = 6)	[−100, 100]	2000
Composition	F20	Composition Function 1 (N = 3)	[−100, 100]	2100
	F21	Composition Function 2 (N = 3)	[−100, 100]	2200
	F22	Composition Function 3 (N = 4)	[−100, 100]	2300
	F23	Composition Function 4 (N = 4)	[−100, 100]	2400
	F24	Composition Function 5 (N = 5)	[−100, 100]	2500
	F25	Composition Function 6 (N = 5)	[−100, 100]	2600
	F26	Composition Function 7 (N = 6)	[−100, 100]	2700
	F27	Composition Function 8 (N = 6)	[−100, 100]	2800
	F28	Composition Function 9 (N = 3)	[−100, 100]	2900
	F29	Composition Function 10 (N = 3)	[−100, 100]	3000

**Table 2:** CEC 2022 test functions.

Type	ID	CEC 2022 Function Name	Search Range	f_min_
Unimodal functions	F1	Shifted and Fully Rotated Zakharov Function	[−100, 100]	300
Multimodal functions	F2	Shifted and Fully Rotated Rosenbrock’s Function	[−100, 100]	400
	F3	Shifted and Fully Rotated Expanded Schaffer’s f6 Function	[−100, 100]	600
	F4	Shifted and Fully Rotated Non-Continuous Rastrigin’s Function	[−100, 100]	800
	F5	Shifted and Fully Rotated Levy Function	[−100, 100]	900
Hybrid functions	F6	Hybrid Function 1 (N = 3)	[−100, 100]	1800
	F7	Hybrid Function 2 (N = 6	[−100, 100]	2000
	F8	Hybrid Function 3 (N = 5)	[−100, 100]	2200
Composition functions	F9	Composition Function 1 (N = 5)	[−100, 100]	2300
	F10	Composition Function 2 (N = 4)	[−100, 100]	2400
	F11	Composition Function 3 (N = 5)	[−100, 100]	2600
	F12	Composition Function 4 (N = 6)	[−100, 100]	2700

**Table 3:** Parameter settings for each algorithm.

Algorithms	References	Parameters
EO	[54]	$a_{1}=2,\;a_{2}=1,\;GP=0.5$
SAO	[55]	$M\in \left(0.35,0.6\right)$
MRFO	[56]	S=2
IYDSE	[57]	β=1.5, L=1, I=0.01, Del=0.38
DFDBARO	[58]	k=1
EOSMA	[59]	$V=1,\;a_{1}=2,\;a_{2}=1,GP=0.5,\;z=0.6,\;q=-0.2$
IGWO	[60]	$a\in \left(0,2\right)$
LSHADE	[61]	H=6, ps=5, ms=5, F=0.5, CR=0.5
RIME	[45]	w=5
HERIME	N/A	w=5, λ=0.5

**Table 4:** Different RIME variants with two strategies.

Strategy	RIME	CRIME	RRIME	HERIME
SFF	NO	YES	NO	YES
RSFDB	NO	NO	YES	YES

**Table 5:** The Friedman test results of RIME, CRIME, RRIME, and HERIME based on CEC 2017 and CEC 2022.

Test Suite	Dimension	RIME	CRIME	RRIME	HERIME	Friedman *p*-Value
CEC 2017	10	3.34	2.00	3.07	1.59	4.76E-08
	30	3.72	1.90	3.28	1.10	1.48E-16
	50	3.83	1.83	3.17	1.17	1.30E-16
	100	3.79	1.69	3.17	1.34	1.93E-15
Mean rank		3.67	1.85	3.17	1.30	N/A
CEC 2022	10	3.42	1.83	3.50	1.25	4.20E-06
	20	3.58	1.92	3.33	1.17	2.59E-06
Mean rank		3.50	1.88	3.42	1.21	N/A
Total mean rank		3.61	1.86	3.25	1.27	N/A

**Table 6:** The Wilcoxon rank sum test results of RIME, CRIME, RRIME, and HERIME based on CEC 2017 and CEC 2022.

vs. RIME +/=/−	CEC 2017 Test Suite				CEC 2022 Test Suite	
	10 D	30 D	50 D	100 D	10 D	20 D
CRIME	23/5/1	29/0/0	29/0/0	29/0/0	9/2/1	12/0/0
RRIME	1/28/0	5/24/0	6/23/0	8/21/0	0/10/2	4/8/0
HERIME	23/3/3	28/1/0	29/0/0	27/2/0	11/1/0	12/0/0

**Table 7:** The Friedman test results of HERIME and competitors in solving CEC2017/CEC2022.

Algorithm	CEC-2017 Test Suite					CEC-2022 Test Suite			Overall Average Ranking
	10 D	30 D	50 D	100 D	Average Ranking	10 D	20 D	Average Ranking	
HERIME	2.38	1.21	1.24	1.86	1.67	2.33	1.42	1.42	1.88
RIME	5.41	5.00	4.69	5.00	5.03	5.50	4.83	4.83	5.17
EO	3.76	2.52	2.45	2.07	2.70	2.92	2.67	2.67	2.79
SAO	6.97	7.52	7.69	7.97	7.53	5.75	7.08	7.08	6.42
MRFO	5.48	5.28	5.86	6.07	5.67	6.25	5.58	5.58	5.92
IYDSE	8.41	8.97	8.72	8.52	8.66	8.83	9.33	9.33	9.08
DFDBARO	6.79	6.90	6.24	5.24	6.29	7.00	7.17	7.17	7.08
EOSMA	2.69	3.34	3.62	3.48	3.28	3.50	3.17	3.17	3.33
IGWO	5.34	5.00	5.21	5.41	5.24	5.58	4.92	4.92	5.25
LSHADE	7.76	9.28	9.28	9.38	8.92	7.33	8.83	8.83	8.08
Friedman *p*-value	1.02E-21	2.92E-39	2.67E-37	4.81E-36	N/A	1.17E-07	9.33E-14	N/A	N/A

**Table 8:** The Wilcoxon rank sum test results of HERIME and competitors in solving CEC2017/CEC2022.

HERIME vs. +/=/−	CEC-2017 Test Suite				CEC-2022 Test Suite	
	10 D	30 D	50 D	100 D	10 D	20 D
RIME	23/3/3	28/1/0	29/0/0	27/2/0	11/1/0	12/0/0
EO	12/14/3	23/6/0	24/4/1	17/4/8	3/4/5	7/4/1
SAO	26/2/1	28/0/1	28/0/1	28/0/1	10/1/1	12/0/0
MRFO	24/3/2	28/0/1	29/0/0	27/1/1	11/0/1	11/0/1
IYDSE	26/2/1	29/0/0	29/0/0	28/0/1	12/0/0	12/0/0
DFDBARO	25/2/2	28/1/0	28/1/0	26/0/3	11/0/1	11/0/1
EOSMA	20/4/5	27/1/1	27/2/0	24/4/1	8/3/1	11/1/0
IGWO	25/2/2	28/1/0	28/1/0	26/1/2	11/0/1	12/0/0
LSHADE	26/1/2	28/0/1	27/0/2	27/0/2	10/1/1	11/0/1

## Data Availability

The data is provided within the manuscript.

## Appendix A

**Table A1:** The experimental results on of HERIME and other competitors based on CEC2017 (10D).

No.	Metric	HERIME	RIME	EO	SAO	MRFO	IYDSE	DFDBARO	EOSMA	IGWO	LSHADE
F1	Best	2.3766E+02	6.8165E+04	1.1341E+02	2.3288E+06	7.0535E+04	9.2785E+07	7.5670E+05	1.4590E+04	4.4611E+05	1.5164E+07
	Ave	3.8835E+03	6.4651E+05	2.6719E+03	7.3548E+06	2.9746E+05	3.6401E+08	7.2029E+06	1.0033E+05	2.1788E+06	1.2164E+08
	Std	3.2515E+03	4.8096E+05	2.7618E+03	4.3879E+06	1.5949E+05	1.9225E+08	5.5314E+06	1.2103E+05	2.0117E+06	6.9658E+07
	Rank	2	5	1	8	4	10	7	3	6	9
F2	Best	3.0000E+02	3.1626E+02	3.0885E+02	4.6849E+02	3.1561E+02	9.3549E+02	3.2601E+03	3.7661E+02	3.1519E+02	6.3102E+03
	Ave	3.0006E+02	3.7721E+02	3.7841E+02	1.1774E+03	4.8918E+02	2.1062E+03	7.8840E+03	8.1120E+02	4.5526E+02	2.2013E+04
	Std	4.5508E-02	5.8413E+01	7.7106E+01	5.9406E+02	2.2877E+02	7.4207E+02	2.2002E+03	3.2568E+02	1.1347E+02	6.5458E+03
	Rank	1	2	3	7	5	8	9	6	4	10
F3	Best	4.0224E+02	4.0068E+02	4.0036E+02	4.0617E+02	4.0122E+02	4.1804E+02	4.0449E+02	4.0436E+02	4.0392E+02	4.0775E+02
	Ave	4.0474E+02	4.1283E+02	4.0695E+02	4.0778E+02	4.0662E+02	4.4381E+02	4.4254E+02	4.0665E+02	4.0775E+02	4.1565E+02
	Std	9.4010E-01	2.0327E+01	1.0782E+00	4.6267E-01	3.0829E+00	1.7668E+01	2.5600E+01	8.1067E-01	2.0338E+00	5.9836E+00
	Rank	1	7	4	6	2	10	9	3	5	8
F4	Best	5.0305E+02	5.0445E+02	5.0349E+02	5.1777E+02	5.1017E+02	5.3308E+02	5.1510E+02	5.0499E+02	5.0576E+02	5.2928E+02
	Ave	5.1165E+02	5.1582E+02	5.1184E+02	5.3407E+02	5.2456E+02	5.4486E+02	5.3381E+02	5.1383E+02	5.2395E+02	5.4130E+02
	Std	5.4865E+00	6.3255E+00	4.7536E+00	7.3028E+00	1.0146E+01	7.0962E+00	7.7533E+00	4.0874E+00	1.0748E+01	5.7012E+00
	Rank	1	4	2	8	6	10	7	3	5	9
F5	Best	6.0002E+02	6.0044E+02	6.0001E+02	6.0099E+02	6.0047E+02	6.1658E+02	6.0182E+02	6.0007E+02	6.0042E+02	6.0112E+02
	Ave	6.0006E+02	6.0123E+02	6.0003E+02	6.0202E+02	6.0165E+02	6.2780E+02	6.0588E+02	6.0032E+02	6.0104E+02	6.0567E+02
	Std	3.6548E-02	7.4787E-01	3.6224E-02	5.3450E-01	1.2065E+00	6.9478E+00	2.6924E+00	1.6296E-01	5.3801E-01	2.6026E+00
	Rank	2	5	1	7	6	10	9	3	4	8
F6	Best	7.0528E+02	7.1712E+02	7.0911E+02	7.3662E+02	7.2395E+02	7.4144E+02	7.2911E+02	7.1576E+02	7.1590E+02	7.3647E+02
	Ave	7.2279E+02	7.3136E+02	7.2103E+02	7.5171E+02	7.5083E+02	7.6642E+02	7.4815E+02	7.2775E+02	7.4164E+02	7.5499E+02
	Std	6.1402E+00	7.8951E+00	5.1758E+00	7.5122E+00	1.3825E+01	1.2296E+01	1.0411E+01	5.4980E+00	9.0590E+00	7.9892E+00
	Rank	2	4	1	8	7	10	6	3	5	9
F7	Best	8.0398E+02	8.0726E+02	8.0501E+02	8.1009E+02	8.0780E+02	8.2550E+02	8.2211E+02	8.0526E+02	8.0364E+02	8.1965E+02
	Ave	8.1102E+02	8.1921E+02	8.1068E+02	8.2439E+02	8.2055E+02	8.3764E+02	8.3156E+02	8.1416E+02	8.2169E+02	8.4095E+02
	Std	5.0364E+00	7.0389E+00	3.8911E+00	8.0449E+00	7.1725E+00	5.2563E+00	6.2937E+00	4.7740E+00	1.0797E+01	6.8879E+00
	Rank	2	4	1	7	5	9	8	3	6	10
F8	Best	9.0000E+02	9.0019E+02	9.0000E+02	9.0040E+02	9.0009E+02	9.5304E+02	9.2391E+02	9.0000E+02	9.0027E+02	9.0298E+02
	Ave	9.0001E+02	9.0130E+02	9.0006E+02	9.0149E+02	9.0333E+02	1.1190E+03	1.0007E+03	9.0037E+02	9.0247E+02	9.6792E+02
	Std	6.3559E-02	1.3001E+00	1.1581E-01	7.6177E-01	5.5307E+00	1.1273E+02	4.4096E+01	4.2678E-01	5.3790E+00	4.8785E+01
	Rank	1	4	2	5	7	10	9	3	6	8
F9	Best	1.0004E+03	1.0248E+03	1.0149E+03	1.2828E+03	1.2862E+03	1.6695E+03	1.4593E+03	1.2729E+03	1.0906E+03	1.9930E+03
	Ave	1.5736E+03	1.5433E+03	1.4917E+03	2.0025E+03	1.9691E+03	2.4527E+03	1.7630E+03	1.8993E+03	2.1200E+03	2.5808E+03
	Std	2.5771E+02	2.7190E+02	2.1583E+02	3.9818E+02	3.6291E+02	2.3179E+02	1.4452E+02	2.7250E+02	5.4337E+02	1.8966E+02
	Rank	3	2	1	7	6	9	4	5	8	10
F10	Best	1.1017E+03	1.1053E+03	1.1007E+03	1.1133E+03	1.1043E+03	1.1607E+03	1.1229E+03	1.1024E+03	1.1037E+03	1.1176E+03
	Ave	1.1060E+03	1.1199E+03	1.1069E+03	1.1231E+03	1.1198E+03	1.2605E+03	1.1790E+03	1.1099E+03	1.1188E+03	1.1628E+03
	Std	3.1468E+00	1.2898E+01	3.9904E+00	4.9585E+00	8.2133E+00	5.2129E+01	3.3892E+01	6.9151E+00	7.5895E+00	2.4934E+01
	Rank	1	6	2	7	5	10	9	3	4	8
F11	Best	1.2545E+03	4.0873E+03	3.1202E+03	8.5708E+04	9.4365E+03	8.2102E+05	5.3088E+04	1.0069E+04	1.0236E+04	2.0633E+06
	Ave	1.6394E+03	1.9818E+06	1.9581E+04	6.1787E+05	2.7483E+05	4.4256E+06	1.0686E+06	1.3040E+05	4.7515E+05	1.3481E+07
	Std	1.9224E+02	2.2569E+06	2.1590E+04	7.8166E+05	7.5625E+05	2.4239E+06	1.0615E+06	1.4992E+05	5.0372E+05	9.8797E+06
	Rank	1	8	2	6	4	9	7	3	5	10
F12	Best	1.3055E+03	1.4571E+03	1.3961E+03	2.4895E+03	1.5203E+03	2.9139E+03	1.6434E+03	1.5617E+03	1.8056E+03	2.0350E+03
	Ave	1.3160E+03	1.1847E+04	6.8989E+03	1.2601E+04	6.3399E+03	8.6300E+03	3.0902E+03	2.3343E+03	7.7816E+03	8.7437E+03
	Std	5.2430E+00	1.0518E+04	7.1702E+03	8.7692E+03	5.1648E+03	4.1442E+03	1.8387E+03	4.6973E+02	5.4531E+03	5.5676E+03
	Rank	1	9	5	10	4	7	3	2	6	8
F13	Best	1.4042E+03	1.4295E+03	1.4494E+03	1.4642E+03	1.4361E+03	1.4498E+03	1.4371E+03	1.4349E+03	1.4546E+03	1.4332E+03
	Ave	1.4179E+03	4.0338E+03	1.5367E+03	2.4946E+03	1.5828E+03	1.5389E+03	1.4645E+03	1.4636E+03	1.5323E+03	1.4564E+03
	Std	7.2635E+00	4.1641E+03	1.0457E+02	2.0560E+03	1.8554E+02	4.2498E+01	1.4641E+01	1.4358E+01	7.0059E+01	1.5899E+01
	Rank	1	10	6	9	8	7	4	3	5	2
F14	Best	1.5010E+03	1.5178E+03	1.5545E+03	1.6511E+03	1.5875E+03	1.7041E+03	1.5684E+03	1.5749E+03	1.5728E+03	1.6067E+03
	Ave	1.5039E+03	5.1328E+03	2.3236E+03	2.4887E+03	2.7561E+03	2.3476E+03	1.7443E+03	1.6627E+03	2.1730E+03	1.9129E+03
	Std	1.1905E+00	5.1226E+03	9.5508E+02	1.0937E+03	1.2127E+03	5.2483E+02	1.1072E+02	7.1929E+01	8.3284E+02	2.2816E+02
	Rank	1	10	6	8	9	7	3	2	5	4
F15	Best	1.6009E+03	1.6018E+03	1.6014E+03	1.6092E+03	1.6053E+03	1.6786E+03	1.6139E+03	1.6031E+03	1.6065E+03	1.6486E+03
	Ave	1.6627E+03	1.7087E+03	1.6416E+03	1.6563E+03	1.7448E+03	1.8729E+03	1.7741E+03	1.6255E+03	1.6512E+03	1.7616E+03
	Std	6.9959E+01	1.0996E+02	5.6103E+01	4.7599E+01	1.0625E+02	7.7796E+01	8.7159E+01	2.7169E+01	5.8690E+01	5.8294E+01
	Rank	5	6	2	4	7	10	9	1	3	8
F16	Best	1.7026E+03	1.7227E+03	1.7229E+03	1.7350E+03	1.7260E+03	1.7501E+03	1.7246E+03	1.7305E+03	1.7350E+03	1.7515E+03
	Ave	1.7390E+03	1.7553E+03	1.7416E+03	1.7587E+03	1.7513E+03	1.7748E+03	1.7590E+03	1.7442E+03	1.7632E+03	1.7814E+03
	Std	2.6072E+01	3.2643E+01	1.5833E+01	1.4108E+01	1.6720E+01	1.1418E+01	2.4484E+01	1.3147E+01	1.8004E+01	1.6157E+01
	Rank	1	5	2	6	4	9	7	3	8	10
F17	Best	1.8086E+03	2.4534E+03	2.0278E+03	5.1840E+03	2.8923E+03	6.5128E+03	2.2779E+03	2.2854E+03	3.4116E+03	2.9007E+03
	Ave	1.8209E+03	1.7731E+04	2.4181E+04	2.8819E+04	1.3523E+04	2.7688E+04	7.2549E+03	5.3216E+03	1.5966E+04	1.8675E+04
	Std	4.5231E+00	1.2999E+04	1.5191E+04	1.4554E+04	8.8820E+03	1.8642E+04	4.1104E+03	3.0925E+03	9.5128E+03	1.2090E+04
	Rank	1	6	8	10	4	9	3	2	5	7
F18	Best	1.9015E+03	1.9085E+03	1.9134E+03	1.9826E+03	1.9564E+03	2.0758E+03	1.9239E+03	1.9200E+03	1.9320E+03	1.9077E+03
	Ave	1.9042E+03	5.3016E+03	5.7252E+03	9.0086E+03	3.7882E+03	3.3352E+03	2.0903E+03	2.0019E+03	3.0313E+03	1.9529E+03
	Std	1.1375E+00	4.8041E+03	6.1508E+03	8.0724E+03	2.5021E+03	1.4709E+03	4.2186E+02	6.7816E+01	1.4169E+03	4.3062E+01
	Rank	1	8	9	10	7	6	4	3	5	2
F19	Best	2.0018E+03	2.0065E+03	2.0017E+03	2.0281E+03	2.0131E+03	2.0875E+03	2.0195E+03	2.0240E+03	2.0248E+03	2.0296E+03
	Ave	2.0269E+03	2.0306E+03	2.0267E+03	2.0480E+03	2.0589E+03	2.1652E+03	2.0454E+03	2.0376E+03	2.0543E+03	2.0654E+03
	Std	1.1364E+01	1.2992E+01	1.1201E+01	1.8696E+01	2.4695E+01	4.4340E+01	1.3466E+01	9.4605E+00	1.5534E+01	1.5853E+01
	Rank	2	3	1	6	8	10	5	4	7	9
F20	Best	2.2000E+03	2.2004E+03	2.2006E+03	2.2019E+03	2.2014E+03	2.2112E+03	2.1367E+03	2.2010E+03	2.2007E+03	2.2213E+03
	Ave	2.2919E+03	2.2776E+03	2.2946E+03	2.2640E+03	2.2573E+03	2.2399E+03	2.2605E+03	2.2525E+03	2.2943E+03	2.3278E+03
	Std	4.5938E+01	5.6035E+01	4.0294E+01	6.5048E+01	5.8464E+01	2.4128E+01	4.2849E+01	5.4849E+01	5.4646E+01	3.1908E+01
	Rank	7	6	9	5	3	1	4	2	8	10
F21	Best	2.2152E+03	2.2289E+03	2.2128E+03	2.2291E+03	2.2006E+03	2.3262E+03	2.2437E+03	2.2117E+03	2.3052E+03	2.3092E+03
	Ave	2.3006E+03	2.3050E+03	2.2992E+03	2.3076E+03	2.2997E+03	2.3825E+03	2.3241E+03	2.2981E+03	2.3089E+03	2.3250E+03
	Std	1.2259E+01	1.5444E+01	1.2345E+01	1.5054E+01	2.6407E+01	2.5760E+01	4.6889E+01	2.0710E+01	9.8756E-01	1.6869E+01
	Rank	4	5	2	6	3	10	8	1	7	9
F22	Best	2.6000E+03	2.3011E+03	2.6003E+03	2.6109E+03	2.6090E+03	2.6261E+03	2.4959E+03	2.6030E+03	2.6051E+03	2.6360E+03
	Ave	2.6139E+03	2.6142E+03	2.6150E+03	2.6278E+03	2.6310E+03	2.6525E+03	2.6459E+03	2.6140E+03	2.6283E+03	2.6523E+03
	Std	5.8518E+00	4.5327E+01	6.7572E+00	8.8982E+00	1.1354E+01	9.1090E+00	2.4226E+01	5.9090E+00	1.2046E+01	6.9379E+00
	Rank	1	3	4	5	7	10	8	2	6	9
F23	Best	2.5001E+03	2.5014E+03	2.5013E+03	2.5041E+03	2.5019E+03	2.5502E+03	2.5135E+03	2.5100E+03	2.5047E+03	2.6439E+03
	Ave	2.7380E+03	2.7327E+03	2.7316E+03	2.7362E+03	2.7110E+03	2.7088E+03	2.6455E+03	2.6705E+03	2.7355E+03	2.7783E+03
	Std	3.4450E+01	6.8515E+01	4.6830E+01	7.7575E+01	8.9174E+01	7.1026E+01	6.0109E+01	9.5933E+01	5.8362E+01	2.2644E+01
	Rank	9	6	5	8	4	3	1	2	7	10
F24	Best	2.8977E+03	2.8979E+03	2.8980E+03	2.9004E+03	2.8980E+03	2.9332E+03	2.8192E+03	2.8983E+03	2.8987E+03	2.9407E+03
	Ave	2.9329E+03	2.9301E+03	2.9357E+03	2.9366E+03	2.9262E+03	2.9720E+03	2.9372E+03	2.9143E+03	2.9344E+03	2.9586E+03
	Std	2.0567E+01	2.4903E+01	1.9797E+01	1.9886E+01	2.7718E+01	1.4301E+01	3.3554E+01	1.9538E+01	1.9025E+01	6.9558E+00
	Rank	4	3	6	7	2	10	8	1	5	9
F25	Best	2.9000E+03	2.8306E+03	2.8002E+03	2.9068E+03	2.8131E+03	3.0187E+03	2.8969E+03	2.9004E+03	2.6333E+03	3.0754E+03
	Ave	2.9119E+03	2.9311E+03	2.9280E+03	2.9343E+03	2.9295E+03	3.2282E+03	3.1421E+03	2.9060E+03	2.9001E+03	3.3190E+03
	Std	2.7374E+01	4.6805E+01	4.2541E+01	2.0584E+01	1.3519E+02	1.1264E+02	1.1954E+02	1.1312E+01	4.1324E+01	1.3981E+02
	Rank	3	6	4	7	5	9	8	2	1	10
F26	Best	3.0890E+03	3.0894E+03	3.0893E+03	3.0903E+03	3.0951E+03	3.1005E+03	3.1107E+03	3.0898E+03	3.0899E+03	3.0765E+03
	Ave	3.0911E+03	3.0960E+03	3.0916E+03	3.0928E+03	3.1107E+03	3.1056E+03	3.1273E+03	3.0920E+03	3.0956E+03	3.0832E+03
	Std	2.0535E+00	3.2717E+00	2.1794E+00	2.2757E+00	2.4243E+01	2.5503E+00	9.5858E+00	2.1786E+00	2.4261E+00	6.9779E+00
	Rank	2	7	3	5	9	8	10	4	6	1
F27	Best	3.1001E+03	3.1033E+03	3.1665E+03	3.1432E+03	2.8253E+03	3.1959E+03	3.1603E+03	3.1038E+03	3.1026E+03	3.2739E+03
	Ave	3.2714E+03	3.2704E+03	3.3030E+03	3.3070E+03	3.2484E+03	3.2744E+03	3.3213E+03	3.1638E+03	3.2487E+03	3.2788E+03
	Std	1.4650E+02	1.2157E+02	1.1326E+02	1.0970E+02	1.4816E+02	3.3017E+01	8.4016E+01	4.4439E+01	1.1534E+02	3.6955E+00
	Rank	5	4	8	9	2	6	10	1	3	7
F28	Best	3.1390E+03	3.1367E+03	3.1393E+03	3.1677E+03	3.1499E+03	3.2044E+03	3.1902E+03	3.1494E+03	3.1479E+03	3.1843E+03
	Ave	3.1671E+03	3.2018E+03	3.1721E+03	3.2126E+03	3.2206E+03	3.2720E+03	3.2533E+03	3.1756E+03	3.1933E+03	3.2959E+03
	Std	1.9334E+01	3.9182E+01	2.2498E+01	2.9096E+01	4.4160E+01	3.6464E+01	3.7691E+01	1.5155E+01	2.5213E+01	4.6139E+01
	Rank	1	5	2	6	7	9	8	3	4	10
F29	Best	3.4213E+03	4.0595E+03	6.1718E+03	6.4111E+03	1.4484E+04	6.7439E+04	4.8368E+04	4.2179E+03	4.8733E+03	3.6914E+03
	Ave	2.6995E+05	3.0991E+05	5.5445E+05	3.7113E+05	8.8983E+05	8.8906E+05	9.8824E+05	7.9893E+04	4.1842E+05	1.8631E+04
	Std	4.2789E+05	4.8412E+05	5.4866E+05	4.8574E+05	1.0793E+06	5.9218E+05	7.9276E+05	1.6166E+05	6.3219E+05	1.4927E+04
	Rank	3	4	7	5	9	8	10	2	6	1

**Table A2:** The experimental results on of HERIME and other competitors based on CEC2017 (30D).

No.	Metric	HERIME	RIME	EO	SAO	MRFO	IYDSE	DFDBARO	EOSMA	IGWO	LSHADE
F1	Best	2.4716E+04	1.1095E+07	4.4941E+04	8.7609E+08	2.2510E+07	2.0831E+09	7.1188E+06	5.4109E+06	3.9747E+07	6.0420E+08
	Ave	1.7212E+05	2.5976E+07	3.1678E+05	1.7789E+09	6.7977E+07	6.3128E+09	2.5506E+07	1.6337E+07	3.6360E+08	7.9938E+09
	Std	1.0748E+05	1.2716E+07	4.1750E+05	3.9273E+08	2.8618E+07	2.3063E+09	1.2662E+07	9.1910E+06	2.8930E+08	6.6824E+09
	Rank	1	5	2	8	6	9	4	3	7	10
F2	Best	3.1626E+02	1.5442E+04	1.7201E+04	4.9903E+04	1.4305E+04	3.2812E+04	5.2431E+04	2.1892E+04	1.3369E+04	1.1999E+05
	Ave	4.7669E+02	3.1608E+04	3.1603E+04	6.5788E+04	2.9885E+04	4.5125E+04	8.0613E+04	3.9741E+04	2.8104E+04	2.1163E+05
	Std	1.4650E+02	9.3060E+03	7.5899E+03	8.8159E+03	6.4852E+03	6.4163E+03	8.7589E+03	8.8744E+03	8.6722E+03	3.3126E+04
	Rank	1	5	4	8	3	7	9	6	2	10
F3	Best	4.8651E+02	4.9075E+02	4.7993E+02	5.9171E+02	4.9728E+02	8.1159E+02	5.2678E+02	4.6878E+02	5.0621E+02	5.8450E+02
	Ave	4.9495E+02	5.2728E+02	5.1326E+02	6.5353E+02	5.6333E+02	1.2561E+03	6.5453E+02	5.2749E+02	5.5739E+02	1.4673E+03
	Std	9.3183E+00	2.3068E+01	1.5071E+01	3.5326E+01	3.0187E+01	2.6643E+02	7.4509E+01	1.8447E+01	2.8489E+01	8.7029E+02
	Rank	1	3	2	7	6	9	8	4	5	10
F4	Best	5.2414E+02	5.4007E+02	5.4344E+02	6.9125E+02	5.9498E+02	6.9709E+02	6.2385E+02	5.5997E+02	5.5653E+02	6.9341E+02
	Ave	5.4910E+02	5.8776E+02	5.7038E+02	7.3338E+02	6.6434E+02	7.5280E+02	6.7276E+02	5.9929E+02	6.5035E+02	7.7530E+02
	Std	1.6093E+01	2.2987E+01	1.4844E+01	1.5453E+01	3.9158E+01	2.6045E+01	2.6090E+01	1.7109E+01	5.5495E+01	3.7172E+01
	Rank	1	3	2	8	6	9	7	4	5	10
F5	Best	6.0013E+02	6.0243E+02	6.0020E+02	6.1446E+02	6.0725E+02	6.4911E+02	6.0640E+02	6.0152E+02	6.0218E+02	6.0716E+02
	Ave	6.0031E+02	6.0883E+02	6.0065E+02	6.1973E+02	6.2337E+02	6.6251E+02	6.1512E+02	6.0302E+02	6.0490E+02	6.3741E+02
	Std	1.3956E-01	3.8238E+00	2.9075E-01	2.7104E+00	1.0015E+01	7.5296E+00	4.4600E+00	9.9322E-01	1.6898E+00	1.5982E+01
	Rank	1	5	2	7	8	10	6	3	4	9
F6	Best	7.5928E+02	8.1652E+02	7.8820E+02	9.6217E+02	9.0550E+02	9.5835E+02	8.5482E+02	8.0741E+02	8.1542E+02	9.7397E+02
	Ave	8.1364E+02	8.9159E+02	8.1686E+02	1.0057E+03	9.8947E+02	1.0748E+03	9.5131E+02	8.5111E+02	9.0630E+02	1.1424E+03
	Std	2.6856E+01	4.0476E+01	1.9449E+01	1.6495E+01	4.9658E+01	6.0149E+01	2.9711E+01	2.2447E+01	4.2390E+01	1.7671E+02
	Rank	1	4	2	8	7	9	6	3	5	10
F7	Best	8.2363E+02	8.3202E+02	8.3742E+02	9.8955E+02	8.6791E+02	9.7544E+02	9.0199E+02	8.5047E+02	8.2701E+02	1.0206E+03
	Ave	8.4870E+02	8.9160E+02	8.6806E+02	1.0203E+03	9.3972E+02	1.0451E+03	9.6344E+02	8.9251E+02	9.2157E+02	1.0713E+03
	Std	1.4543E+01	2.4289E+01	1.5437E+01	1.4831E+01	3.3643E+01	2.2684E+01	2.8925E+01	1.8428E+01	5.6436E+01	2.5179E+01
	Rank	1	3	2	8	6	9	7	4	5	10
F8	Best	9.0020E+02	9.6620E+02	9.0180E+02	1.3663E+03	1.3046E+03	3.9832E+03	2.8319E+03	9.1429E+02	9.3164E+02	2.1828E+03
	Ave	9.0137E+02	1.8282E+03	9.2861E+02	1.8437E+03	4.1912E+03	6.2648E+03	5.0261E+03	9.9833E+02	1.3170E+03	8.2336E+03
	Std	1.3724E+00	8.2733E+02	2.8562E+01	3.1804E+02	1.7946E+03	1.2300E+03	1.2511E+03	7.9543E+01	2.9979E+02	3.4420E+03
	Rank	1	5	2	6	7	9	8	3	4	10
F9	Best	3.2333E+03	3.1979E+03	3.1515E+03	6.2699E+03	4.0724E+03	7.1420E+03	3.8247E+03	5.1290E+03	3.1402E+03	7.5183E+03
	Ave	4.6069E+03	4.7362E+03	5.0382E+03	8.2990E+03	6.5089E+03	8.1048E+03	4.6344E+03	6.7139E+03	7.9165E+03	8.7614E+03
	Std	5.4352E+02	6.6385E+02	7.8123E+02	4.1105E+02	9.4294E+02	2.9862E+02	4.4226E+02	6.3841E+02	1.2366E+03	3.4847E+02
	Rank	1	3	4	9	5	8	2	6	7	10
F10	Best	1.1214E+03	1.2182E+03	1.1597E+03	1.4213E+03	1.3237E+03	2.0601E+03	1.4400E+03	1.2435E+03	1.2415E+03	2.7376E+03
	Ave	1.1647E+03	1.3488E+03	1.2362E+03	1.6233E+03	1.4251E+03	3.1707E+03	1.8123E+03	1.3305E+03	1.3249E+03	6.8655E+03
	Std	2.9349E+01	6.5266E+01	3.3024E+01	8.6910E+01	5.4779E+01	5.0259E+02	2.4547E+02	4.7988E+01	4.4589E+01	2.3672E+03
	Rank	1	5	2	7	6	9	8	4	3	10
F11	Best	1.1919E+04	1.5041E+06	1.0786E+05	6.6738E+07	4.5070E+05	1.8492E+08	2.1731E+06	3.0257E+05	3.8370E+06	3.1904E+08
	Ave	4.2953E+04	1.9603E+07	1.5830E+06	1.4889E+08	3.7532E+06	6.4005E+08	1.7979E+07	2.3269E+06	2.0768E+07	9.9130E+08
	Std	2.9081E+04	1.6043E+07	1.3967E+06	4.0475E+07	2.3603E+06	2.2109E+08	1.1156E+07	1.5070E+06	1.4484E+07	5.3138E+08
	Rank	1	6	2	8	4	9	5	3	7	10
F12	Best	1.9224E+03	6.6832E+04	3.7442E+03	9.3988E+06	8.0326E+03	2.3790E+07	8.0931E+04	1.7187E+04	1.1909E+05	7.6351E+06
	Ave	4.1248E+03	3.0218E+05	3.1401E+04	4.1556E+07	3.4113E+04	9.6066E+07	7.6965E+05	5.8109E+04	4.6381E+05	8.3825E+07
	Std	2.9884E+03	3.4157E+05	2.3208E+04	2.0190E+07	2.4724E+04	5.6356E+07	7.0211E+05	4.4934E+04	2.4318E+05	6.7097E+07
	Rank	1	5	2	8	3	10	7	4	6	9
F13	Best	1.4459E+03	1.9731E+03	3.1300E+03	2.5442E+04	2.5342E+03	1.3730E+04	4.0349E+03	2.2862E+03	1.9962E+03	7.8305E+04
	Ave	1.4731E+03	4.9690E+04	4.2458E+04	1.0979E+05	3.3332E+04	8.8497E+04	8.4235E+04	6.6706E+03	2.7459E+04	5.0406E+05
	Std	1.2973E+01	3.7987E+04	4.3833E+04	6.3707E+04	3.4940E+04	3.9670E+04	1.3016E+05	4.5084E+03	2.2422E+04	2.7166E+05
	Rank	1	6	5	9	4	8	7	2	3	10
F14	Best	1.5761E+03	1.6329E+04	1.8048E+03	8.4916E+04	2.0610E+03	7.9526E+04	1.2484E+04	3.2276E+03	1.6743E+04	8.2999E+05
	Ave	1.6745E+03	6.9438E+04	6.5554E+03	5.9646E+05	9.5820E+03	1.1828E+06	5.1679E+04	3.2308E+04	6.1183E+04	9.3025E+06
	Std	5.6615E+01	3.5734E+04	5.6724E+03	3.6493E+05	8.2139E+03	7.0731E+05	3.8691E+04	2.3610E+04	3.5059E+04	7.1045E+06
	Rank	1	7	2	8	3	9	5	4	6	10
F15	Best	1.6757E+03	1.8116E+03	1.7787E+03	2.5139E+03	2.1796E+03	3.3913E+03	2.5808E+03	1.8633E+03	1.8800E+03	3.3242E+03
	Ave	2.3095E+03	2.5873E+03	2.2673E+03	3.3119E+03	2.7869E+03	3.7319E+03	2.9971E+03	2.4785E+03	2.6705E+03	3.9159E+03
	Std	2.7742E+02	2.7960E+02	3.2103E+02	2.9369E+02	2.8995E+02	1.6350E+02	2.2076E+02	2.8913E+02	5.3992E+02	2.8519E+02
	Rank	2	4	1	8	6	9	7	3	5	10
F16	Best	1.7479E+03	1.7891E+03	1.7579E+03	1.8922E+03	1.7934E+03	2.1613E+03	2.0026E+03	1.7912E+03	1.7966E+03	2.3873E+03
	Ave	1.9154E+03	2.1237E+03	1.9516E+03	2.1663E+03	2.0999E+03	2.4072E+03	2.3677E+03	1.8976E+03	1.9789E+03	2.8121E+03
	Std	1.2969E+02	1.7589E+02	1.6482E+02	2.1544E+02	2.2487E+02	1.0305E+02	1.5562E+02	7.2108E+01	1.6703E+02	1.8134E+02
	Rank	2	6	3	7	5	9	8	1	4	10
F17	Best	1.8553E+03	8.8597E+04	3.4766E+04	2.6283E+05	8.7241E+04	1.5708E+05	7.0541E+04	3.8522E+04	5.4602E+04	1.6089E+06
	Ave	1.9246E+03	1.5223E+06	6.1037E+05	1.3946E+06	5.6505E+05	6.3596E+05	7.0847E+05	2.2219E+05	5.7634E+05	1.3125E+07
	Std	4.7883E+01	1.3842E+06	6.2822E+05	6.9858E+05	5.2182E+05	2.5976E+05	5.2060E+05	1.4966E+05	5.4483E+05	5.8421E+06
	Rank	1	9	5	8	3	6	7	2	4	10
F18	Best	1.9267E+03	1.1328E+04	1.9694E+03	1.9318E+05	2.1048E+03	8.2268E+05	8.9234E+03	2.6130E+03	1.4270E+04	1.0181E+06
	Ave	1.9521E+03	3.0352E+05	1.0356E+04	2.0710E+06	1.0285E+04	2.7549E+06	5.7647E+04	2.0441E+04	1.6992E+05	1.7778E+07
	Std	1.6802E+01	2.6260E+05	1.1046E+04	1.5071E+06	1.0695E+04	1.2974E+06	6.4465E+04	2.4252E+04	2.5398E+05	1.7284E+07
	Rank	1	7	3	8	2	9	5	4	6	10
F19	Best	2.0331E+03	2.0951E+03	2.0388E+03	2.1960E+03	2.2504E+03	2.4964E+03	2.1976E+03	2.1423E+03	2.1197E+03	2.5525E+03
	Ave	2.2381E+03	2.4037E+03	2.2601E+03	2.4130E+03	2.4968E+03	2.7256E+03	2.5372E+03	2.3104E+03	2.3947E+03	2.9213E+03
	Std	1.4331E+02	1.3425E+02	1.2844E+02	1.3591E+02	1.5177E+02	1.0626E+02	1.4861E+02	8.1705E+01	1.7331E+02	1.1508E+02
	Rank	1	5	2	6	7	9	8	3	4	10
F20	Best	2.3245E+03	2.3469E+03	2.3306E+03	2.4661E+03	2.3750E+03	2.5188E+03	2.4097E+03	2.3548E+03	2.3493E+03	2.5220E+03
	Ave	2.3476E+03	2.3949E+03	2.3639E+03	2.5129E+03	2.4300E+03	2.5652E+03	2.4803E+03	2.3931E+03	2.4255E+03	2.5703E+03
	Std	1.2191E+01	2.4747E+01	1.6096E+01	1.5252E+01	2.9660E+01	2.2256E+01	3.1124E+01	1.5287E+01	4.9878E+01	3.0446E+01
	Rank	1	4	2	8	6	9	7	3	5	10
F21	Best	2.3015E+03	2.3205E+03	2.3032E+03	2.4880E+03	2.3302E+03	3.4603E+03	2.3244E+03	2.3163E+03	2.3388E+03	8.4354E+03
	Ave	2.9405E+03	4.0308E+03	3.0155E+03	2.5833E+03	2.4394E+03	4.3429E+03	4.4354E+03	2.3230E+03	3.0172E+03	9.9192E+03
	Std	1.4023E+03	2.0062E+03	1.5679E+03	4.1384E+01	6.2329E+02	5.8348E+02	8.4224E+02	4.7846E+00	1.8419E+03	4.5612E+02
	Rank	4	7	5	3	2	8	9	1	6	10
F22	Best	2.6677E+03	2.7179E+03	2.6673E+03	2.8577E+03	2.7551E+03	2.9196E+03	2.7816E+03	2.7089E+03	2.7083E+03	2.8854E+03
	Ave	2.7003E+03	2.7487E+03	2.7097E+03	2.8856E+03	2.8439E+03	2.9834E+03	2.8978E+03	2.7424E+03	2.7874E+03	2.9613E+03
	Std	1.4913E+01	1.8741E+01	1.5358E+01	1.5362E+01	4.5943E+01	2.5013E+01	4.9062E+01	2.2076E+01	4.9878E+01	4.2538E+01
	Rank	1	4	2	7	6	10	8	3	5	9
F23	Best	2.8387E+03	2.8735E+03	2.8427E+03	3.0030E+03	2.9153E+03	3.0703E+03	2.9764E+03	2.8584E+03	2.8644E+03	3.0798E+03
	Ave	2.8734E+03	2.9219E+03	2.8742E+03	3.0461E+03	3.0004E+03	3.1402E+03	3.0936E+03	2.8959E+03	2.9518E+03	3.1448E+03
	Std	1.7232E+01	2.9484E+01	1.4128E+01	1.4339E+01	4.4032E+01	2.9090E+01	4.7282E+01	1.9878E+01	6.1862E+01	4.8909E+01
	Rank	1	4	2	7	6	9	8	3	5	10
F24	Best	2.8870E+03	2.8936E+03	2.8848E+03	2.9393E+03	2.9014E+03	3.0628E+03	2.9319E+03	2.8934E+03	2.9029E+03	2.9444E+03
	Ave	2.8878E+03	2.9272E+03	2.9042E+03	2.9773E+03	2.9517E+03	3.2149E+03	2.9921E+03	2.9220E+03	2.9491E+03	3.4356E+03
	Std	1.1428E+00	1.9557E+01	1.3483E+01	1.7881E+01	2.7590E+01	8.9608E+01	3.8418E+01	1.7273E+01	2.0684E+01	3.8304E+02
	Rank	1	4	2	7	6	9	8	3	5	10
F25	Best	3.7843E+03	3.4077E+03	3.9401E+03	5.4387E+03	2.9841E+03	5.9835E+03	4.4004E+03	2.9107E+03	3.4643E+03	5.5872E+03
	Ave	4.1104E+03	4.7620E+03	4.2434E+03	5.9331E+03	5.3558E+03	6.8755E+03	5.5509E+03	4.3712E+03	4.8255E+03	6.4152E+03
	Std	1.4493E+02	3.7029E+02	1.7059E+02	1.7663E+02	1.2140E+03	3.3879E+02	6.5474E+02	4.0217E+02	5.1366E+02	5.0028E+02
	Rank	1	4	2	8	6	10	7	3	5	9
F26	Best	3.1920E+03	3.2109E+03	3.1950E+03	3.2373E+03	3.2458E+03	3.3074E+03	3.2538E+03	3.2078E+03	3.2130E+03	3.2000E+03
	Ave	3.2080E+03	3.2348E+03	3.2135E+03	3.2521E+03	3.3051E+03	3.3718E+03	3.3012E+03	3.2309E+03	3.2352E+03	3.2000E+03
	Std	7.9989E+00	1.4095E+01	9.5738E+00	9.9632E+00	3.0646E+01	3.1386E+01	2.3911E+01	9.5928E+00	1.3279E+01	6.0121E-05
	Rank	2	5	3	7	9	10	8	4	6	1
F27	Best	3.2085E+03	3.2431E+03	3.2074E+03	3.3386E+03	3.2393E+03	3.6053E+03	3.2877E+03	3.2222E+03	3.2577E+03	3.2996E+03
	Ave	3.2340E+03	3.3141E+03	3.2604E+03	3.4088E+03	3.3183E+03	3.8367E+03	3.3584E+03	3.2747E+03	3.3494E+03	3.3000E+03
	Std	1.9762E+01	4.4824E+01	2.7735E+01	2.8988E+01	3.2438E+01	1.4915E+02	4.8690E+01	2.5860E+01	4.7535E+01	5.3478E-02
	Rank	1	5	2	9	6	10	8	3	7	4
F28	Best	3.3943E+03	3.5107E+03	3.3694E+03	3.7931E+03	3.5905E+03	4.4232E+03	3.8484E+03	3.5350E+03	3.4529E+03	4.1334E+03
	Ave	3.5791E+03	3.8222E+03	3.5961E+03	4.2300E+03	4.0143E+03	4.8226E+03	4.3589E+03	3.7434E+03	3.7196E+03	5.0089E+03
	Std	1.2111E+02	1.7091E+02	1.5528E+02	2.3366E+02	2.0148E+02	1.9586E+02	2.0924E+02	1.3080E+02	2.1772E+02	2.4320E+02
	Rank	1	5	2	7	6	9	8	4	3	10
F29	Best	5.7388E+03	5.3146E+05	6.5879E+03	3.4325E+06	3.4353E+04	8.9698E+06	1.7849E+05	4.2506E+04	4.5094E+05	1.0140E+06
	Ave	7.7597E+03	2.1686E+06	2.1427E+04	1.1159E+07	1.8982E+05	2.0658E+07	5.8471E+05	3.4088E+05	1.9522E+06	5.8495E+06
	Std	1.1988E+03	1.4836E+06	1.4841E+04	4.9502E+06	1.8077E+05	8.5768E+06	2.9562E+05	2.5514E+05	1.2206E+06	4.8614E+06
	Rank	1	7	2	9	3	10	5	4	6	8

**Table A3:** The experimental results on of HERIME and other competitors based on CEC2017 (50D).

No.	Metric	HERIME	RIME	EO	SAO	MRFO	IYDSE	DFDBARO	EOSMA	IGWO	LSHADE
F1	Best	4.2798E+05	7.5653E+07	2.8174E+06	4.9408E+09	3.3653E+08	9.4900E+09	8.0685E+07	6.8899E+07	1.8490E+08	4.6912E+09
	Ave	1.5072E+06	1.6687E+08	1.1074E+07	8.3549E+09	8.4519E+08	1.8346E+10	1.6886E+08	1.9175E+08	2.1750E+09	5.1133E+10
	Std	1.0396E+06	6.8565E+07	6.1992E+06	1.5618E+09	2.8341E+08	4.0912E+09	7.3604E+07	1.1131E+08	1.5256E+09	3.4082E+10
	Rank	1	3	2	8	6	9	4	5	7	10
F2	Best	3.3326E+03	6.7807E+04	5.2973E+04	1.0141E+05	7.2765E+04	1.3694E+05	1.5276E+05	6.4191E+04	4.9549E+04	2.9515E+05
	Ave	6.6845E+03	1.0290E+05	9.0964E+04	1.4476E+05	1.3426E+05	1.7568E+05	1.8519E+05	1.1099E+05	8.3111E+04	4.1855E+05
	Std	2.6203E+03	2.2994E+04	2.1342E+04	1.6522E+04	2.2350E+04	1.6762E+04	1.5518E+04	1.9158E+04	1.6000E+04	6.3907E+04
	Rank	1	4	3	7	6	8	9	5	2	10
F3	Best	5.0567E+02	5.8295E+02	4.8342E+02	1.1168E+03	6.6473E+02	1.6832E+03	7.1030E+02	5.3461E+02	6.4784E+02	1.1688E+03
	Ave	5.8084E+02	6.9705E+02	5.9584E+02	1.4304E+03	8.4527E+02	3.1190E+03	8.9494E+02	6.6439E+02	7.8942E+02	9.4490E+03
	Std	3.8946E+01	4.7982E+01	4.2748E+01	1.8601E+02	9.6065E+01	1.0062E+03	1.1005E+02	4.0435E+01	1.1775E+02	7.1404E+03
	Rank	1	4	2	8	6	9	7	3	5	10
F4	Best	5.5621E+02	6.2880E+02	6.0351E+02	9.0724E+02	7.7423E+02	9.3366E+02	7.0362E+02	6.4297E+02	6.4511E+02	9.3761E+02
	Ave	5.9023E+02	7.1217E+02	6.7367E+02	9.7073E+02	8.3146E+02	1.0047E+03	8.1644E+02	7.1248E+02	7.5332E+02	1.1105E+03
	Std	1.8465E+01	4.6598E+01	3.4339E+01	2.2713E+01	3.2961E+01	3.3445E+01	4.3909E+01	3.1599E+01	9.9186E+01	8.7077E+01
	Rank	1	3	2	8	7	9	6	4	5	10
F5	Best	6.0030E+02	6.0729E+02	6.0090E+02	6.2771E+02	6.2255E+02	6.5415E+02	6.0988E+02	6.0372E+02	6.0581E+02	6.1229E+02
	Ave	6.0057E+02	6.1675E+02	6.0233E+02	6.3271E+02	6.4292E+02	6.7698E+02	6.2016E+02	6.0708E+02	6.1113E+02	6.6497E+02
	Std	1.4549E-01	4.4739E+00	1.0106E+00	2.7297E+00	1.2912E+01	7.7888E+00	3.9444E+00	1.9044E+00	3.3599E+00	1.9656E+01
	Rank	1	5	2	7	8	10	6	3	4	9
F6	Best	8.6128E+02	1.0250E+03	8.6953E+02	1.2338E+03	1.0935E+03	1.2655E+03	9.9457E+02	9.2587E+02	9.7853E+02	1.1841E+03
	Ave	9.5271E+02	1.1099E+03	9.5144E+02	1.3207E+03	1.3426E+03	1.4116E+03	1.1875E+03	1.0200E+03	1.0725E+03	1.9854E+03
	Std	4.7521E+01	5.2719E+01	3.4161E+01	3.3551E+01	1.3247E+02	8.0445E+01	6.5472E+01	3.0499E+01	7.1879E+01	6.3527E+02
	Rank	2	5	1	7	8	9	6	3	4	10
F7	Best	8.5003E+02	9.3321E+02	9.1427E+02	1.2185E+03	1.0459E+03	1.2341E+03	1.0331E+03	9.4106E+02	9.3310E+02	1.2613E+03
	Ave	8.9496E+02	1.0174E+03	9.7710E+02	1.2743E+03	1.1354E+03	1.2977E+03	1.1343E+03	1.0143E+03	1.0529E+03	1.4012E+03
	Std	2.0832E+01	4.2139E+01	3.9044E+01	2.2778E+01	4.3634E+01	3.0148E+01	4.2886E+01	3.1428E+01	9.7431E+01	7.9120E+01
	Rank	1	4	2	8	7	9	6	3	5	10
F8	Best	9.0198E+02	2.0331E+03	9.4808E+02	4.3862E+03	9.0794E+03	1.5495E+04	9.4961E+03	1.2179E+03	1.5034E+03	9.0701E+03
	Ave	9.0990E+02	6.5345E+03	1.2529E+03	6.4652E+03	1.8130E+04	2.1494E+04	1.4862E+04	1.8511E+03	4.3029E+03	3.5301E+04
	Std	6.0518E+00	4.1683E+03	2.7403E+02	1.1265E+03	5.2026E+03	3.1097E+03	2.5512E+03	4.8277E+02	1.7924E+03	1.2914E+04
	Rank	1	6	2	5	8	9	7	3	4	10
F9	Best	4.8502E+03	6.4617E+03	5.6571E+03	1.2682E+04	6.9626E+03	1.2914E+04	6.3137E+03	9.2202E+03	7.5512E+03	1.3457E+04
	Ave	7.3898E+03	8.3554E+03	8.8799E+03	1.4263E+04	1.0426E+04	1.3876E+04	7.6498E+03	1.1777E+04	1.4180E+04	1.5070E+04
	Std	1.0645E+03	9.6294E+02	1.3002E+03	5.4312E+02	1.7448E+03	4.2940E+02	6.3014E+02	8.7614E+02	1.7470E+03	5.3442E+02
	Rank	1	3	4	9	5	7	2	6	8	10
F10	Best	1.1919E+03	1.5067E+03	1.3321E+03	3.4650E+03	1.6147E+03	2.9408E+03	2.1867E+03	1.5249E+03	1.3974E+03	1.3149E+04
	Ave	1.2425E+03	1.7071E+03	1.4561E+03	4.9914E+03	1.9188E+03	4.5912E+03	3.3712E+03	1.7827E+03	1.7007E+03	3.8056E+04
	Std	2.7229E+01	1.3540E+02	6.9874E+01	6.8938E+02	2.2026E+02	7.8501E+02	8.8760E+02	1.3807E+02	1.4356E+02	8.7529E+03
	Rank	1	4	2	9	6	8	7	5	3	10
F11	Best	3.6303E+05	3.0014E+07	1.8451E+06	8.4340E+08	1.4182E+07	1.2287E+09	3.3965E+07	8.0010E+06	4.8639E+07	5.1184E+09
	Ave	1.5900E+06	1.7155E+08	9.7668E+06	1.9583E+09	3.9961E+07	3.4163E+09	8.9311E+07	2.8289E+07	2.0134E+08	1.3712E+10
	Std	8.3525E+05	1.0385E+08	5.2352E+06	5.3064E+08	1.7651E+07	1.0919E+09	3.9809E+07	1.6390E+07	1.1120E+08	5.6443E+09
	Rank	1	6	2	8	4	9	5	3	7	10
F12	Best	8.2935E+03	2.3553E+05	9.1034E+03	1.4161E+08	4.5900E+04	1.6122E+08	4.2219E+04	2.7859E+04	8.9476E+05	5.7252E+06
	Ave	1.5705E+04	1.0519E+06	2.8890E+04	3.9904E+08	9.9937E+04	7.6603E+08	2.5772E+06	1.0723E+05	1.6995E+07	6.8937E+08
	Std	7.2099E+03	8.9252E+05	2.6120E+04	1.1507E+08	3.5629E+04	3.0755E+08	2.3517E+06	8.0973E+04	1.8786E+07	6.3953E+08
	Rank	1	5	2	8	3	10	6	4	7	9
F13	Best	1.5101E+03	7.2457E+04	7.0984E+04	3.2722E+05	4.2132E+04	2.1273E+05	4.6608E+04	1.4642E+04	1.1566E+04	2.3224E+06
	Ave	1.5549E+03	3.8413E+05	2.9237E+05	1.3136E+06	2.5826E+05	7.9842E+05	8.6665E+05	7.6525E+04	1.7048E+05	6.7586E+06
	Std	2.5037E+01	2.9802E+05	1.8782E+05	5.6504E+05	2.1355E+05	3.0351E+05	7.8787E+05	4.5216E+04	1.2712E+05	3.0087E+06
	Rank	1	6	5	9	4	7	8	2	3	10
F14	Best	1.8585E+03	4.3849E+04	3.1256E+03	4.8940E+06	4.6535E+03	1.2761E+07	1.6090E+04	7.3130E+03	5.8584E+04	5.9877E+06
	Ave	2.3248E+03	2.4979E+05	1.3248E+04	2.6032E+07	1.3649E+04	3.4662E+07	1.7751E+05	2.6223E+04	7.1362E+05	8.5273E+07
	Std	4.2032E+02	4.2836E+05	7.1595E+03	1.1677E+07	7.2224E+03	1.8250E+07	1.7675E+05	1.8573E+04	1.3477E+06	8.1389E+07
	Rank	1	6	2	8	3	9	5	4	7	10
F15	Best	2.3880E+03	2.3650E+03	2.1362E+03	3.5633E+03	2.5041E+03	4.4539E+03	3.0460E+03	2.0834E+03	2.2460E+03	4.6377E+03
	Ave	3.0870E+03	3.4831E+03	3.0302E+03	5.0479E+03	3.4901E+03	5.0723E+03	4.0008E+03	3.1427E+03	3.0532E+03	6.0308E+03
	Std	3.4196E+02	4.0915E+02	4.3907E+02	4.6978E+02	4.4459E+02	3.0492E+02	3.4686E+02	3.7415E+02	6.4100E+02	4.2971E+02
	Rank	3	5	1	8	6	9	7	4	2	10
F16	Best	2.1889E+03	2.2396E+03	2.0870E+03	3.6405E+03	2.4071E+03	3.2237E+03	3.0556E+03	2.3118E+03	2.1940E+03	3.7995E+03
	Ave	2.7150E+03	3.2127E+03	2.7230E+03	4.1345E+03	3.1818E+03	3.7188E+03	3.5934E+03	2.7660E+03	3.1110E+03	5.1161E+03
	Std	3.0942E+02	2.8149E+02	3.5040E+02	2.0679E+02	3.4410E+02	2.0607E+02	2.2206E+02	2.8265E+02	5.6615E+02	6.0546E+02
	Rank	1	6	2	9	5	8	7	3	4	10
F17	Best	2.0371E+03	2.9798E+05	1.6666E+05	1.9358E+06	2.9673E+05	7.0802E+05	7.5521E+05	1.7186E+05	1.0598E+05	1.6223E+07
	Ave	2.2682E+03	4.1372E+06	2.2706E+06	7.3573E+06	2.3178E+06	2.0708E+06	5.1490E+06	1.0396E+06	1.8673E+06	6.2021E+07
	Std	1.3641E+02	3.5138E+06	1.4019E+06	3.7460E+06	1.6496E+06	8.6902E+05	3.2326E+06	6.5092E+05	1.2986E+06	2.2046E+07
	Rank	1	7	5	9	6	4	8	2	3	10
F18	Best	1.9788E+03	3.6156E+04	3.0597E+03	5.7312E+06	2.8616E+03	4.3620E+06	6.7772E+03	2.8118E+03	6.8645E+04	7.9073E+06
	Ave	2.0467E+03	1.0468E+06	1.8600E+04	2.3263E+07	1.5997E+04	2.0588E+07	8.6663E+04	1.8427E+04	1.1165E+06	5.6876E+07
	Std	4.2832E+01	9.4076E+05	1.1928E+04	1.2440E+07	9.5427E+03	9.3022E+06	1.1212E+05	9.4029E+03	9.2216E+05	4.7588E+07
	Rank	1	6	4	9	2	8	5	3	7	10
F19	Best	2.1491E+03	2.4865E+03	2.3540E+03	2.7729E+03	2.4872E+03	3.2480E+03	2.7369E+03	2.4537E+03	2.2670E+03	3.8160E+03
	Ave	2.7932E+03	3.0657E+03	2.9456E+03	3.6170E+03	3.1351E+03	3.7008E+03	3.3117E+03	2.9925E+03	3.2973E+03	4.3549E+03
	Std	2.5698E+02	2.5773E+02	3.0103E+02	3.3966E+02	2.9848E+02	1.4424E+02	2.4888E+02	2.5058E+02	5.8734E+02	1.8797E+02
	Rank	1	4	2	8	5	9	7	3	6	10
F20	Best	2.3654E+03	2.4230E+03	2.3983E+03	2.6839E+03	2.4654E+03	2.7419E+03	2.5353E+03	2.4377E+03	2.4160E+03	2.7583E+03
	Ave	2.3980E+03	2.5121E+03	2.4544E+03	2.7537E+03	2.5885E+03	2.8515E+03	2.6679E+03	2.4947E+03	2.5405E+03	2.9122E+03
	Std	1.6895E+01	3.9902E+01	2.8319E+01	1.9072E+01	4.8478E+01	4.1350E+01	6.6490E+01	3.2118E+01	8.7534E+01	8.1452E+01
	Rank	1	4	2	8	6	9	7	3	5	10
F21	Best	6.2303E+03	8.1513E+03	8.1018E+03	3.1601E+03	2.4514E+03	1.4809E+04	6.1173E+03	2.4688E+03	2.6404E+03	1.5726E+04
	Ave	8.7873E+03	9.9254E+03	1.0200E+04	7.9981E+03	1.1774E+04	1.5902E+04	9.2826E+03	1.1343E+04	1.3987E+04	1.6575E+04
	Std	1.1272E+03	8.4287E+02	1.0676E+03	5.9346E+03	2.6894E+03	4.3787E+02	9.6619E+02	3.8190E+03	3.9018E+03	4.4253E+02
	Rank	2	4	5	1	7	9	3	6	8	10
F22	Best	2.7749E+03	2.8660E+03	2.7928E+03	3.1599E+03	3.0515E+03	3.3476E+03	3.1155E+03	2.8524E+03	2.8382E+03	3.1258E+03
	Ave	2.8296E+03	2.9632E+03	2.8576E+03	3.2178E+03	3.1708E+03	3.4209E+03	3.2409E+03	2.9249E+03	3.0166E+03	3.4300E+03
	Std	2.8970E+01	5.0175E+01	2.9225E+01	2.7870E+01	6.5231E+01	4.2100E+01	6.6256E+01	2.9998E+01	9.5190E+01	1.7007E+02
	Rank	1	4	2	7	6	9	8	3	5	10
F23	Best	2.9551E+03	3.0207E+03	2.9778E+03	3.2882E+03	3.2133E+03	3.4697E+03	3.2098E+03	3.0005E+03	3.0457E+03	3.3999E+03
	Ave	3.0058E+03	3.1101E+03	3.0204E+03	3.3650E+03	3.3583E+03	3.5413E+03	3.4611E+03	3.0614E+03	3.1772E+03	3.5988E+03
	Std	3.8530E+01	4.5500E+01	2.5861E+01	2.8262E+01	8.6581E+01	3.8886E+01	9.1152E+01	2.7784E+01	1.0388E+02	1.1685E+02
	Rank	1	4	2	7	6	9	8	3	5	10
F24	Best	2.9869E+03	3.0626E+03	3.0659E+03	3.4783E+03	3.1677E+03	4.0411E+03	3.1569E+03	3.1069E+03	3.1410E+03	3.3370E+03
	Ave	3.0460E+03	3.1471E+03	3.1342E+03	3.7391E+03	3.3410E+03	4.8247E+03	3.2308E+03	3.1830E+03	3.2890E+03	1.0077E+04
	Std	1.7621E+01	5.7113E+01	3.6578E+01	1.3358E+02	1.0793E+02	5.1556E+02	3.5690E+01	3.7359E+01	1.0956E+02	4.7905E+03
	Rank	1	3	2	8	7	9	5	4	6	10
F25	Best	4.2877E+03	5.1796E+03	3.3835E+03	8.0334E+03	4.1145E+03	9.3243E+03	6.5366E+03	4.8922E+03	5.0117E+03	7.9299E+03
	Ave	4.6986E+03	6.2100E+03	5.0766E+03	8.6823E+03	7.2894E+03	1.0662E+04	8.3705E+03	5.5216E+03	6.5780E+03	1.0635E+04
	Std	2.5636E+02	5.2048E+02	3.8065E+02	2.3395E+02	2.4619E+03	5.8833E+02	9.8941E+02	3.1282E+02	8.9849E+02	1.5163E+03
	Rank	1	4	2	8	6	10	7	3	5	9
F26	Best	3.2227E+03	3.3646E+03	3.2600E+03	3.4508E+03	3.6491E+03	3.8882E+03	3.6184E+03	3.2960E+03	3.3478E+03	3.2000E+03
	Ave	3.2848E+03	3.4743E+03	3.3442E+03	3.5920E+03	3.8893E+03	4.1244E+03	3.8212E+03	3.4314E+03	3.4478E+03	3.2000E+03
	Std	4.0743E+01	6.8766E+01	5.8263E+01	7.4635E+01	1.3927E+02	1.2265E+02	9.4743E+01	5.8123E+01	6.1692E+01	6.3498E-05
	Rank	2	6	3	7	9	10	8	4	5	1
F27	Best	3.2698E+03	3.3762E+03	3.2817E+03	3.7276E+03	3.5034E+03	4.6174E+03	3.4851E+03	3.4068E+03	3.4807E+03	3.3000E+03
	Ave	3.3202E+03	3.5077E+03	3.4303E+03	4.0469E+03	3.7972E+03	5.5937E+03	3.7800E+03	3.5477E+03	3.8563E+03	3.3000E+03
	Std	2.1912E+01	1.0101E+02	5.2868E+01	1.4359E+02	1.5306E+02	4.5588E+02	6.6586E+02	7.8322E+01	2.4873E+02	6.5747E-05
	Rank	2	4	3	9	7	10	6	5	8	1
F28	Best	3.4019E+03	4.0468E+03	3.4124E+03	4.9867E+03	4.2010E+03	5.7630E+03	4.6921E+03	3.7402E+03	3.9024E+03	6.3140E+03
	Ave	3.8547E+03	4.7130E+03	4.0160E+03	5.8788E+03	4.8938E+03	7.3023E+03	5.3989E+03	4.2519E+03	4.2695E+03	7.8062E+03
	Std	2.1935E+02	3.5740E+02	3.2353E+02	2.6870E+02	3.5368E+02	4.6974E+02	2.8014E+02	2.5479E+02	2.7055E+02	6.7661E+02
	Rank	1	5	2	8	6	9	7	3	4	10
F29	Best	1.1018E+06	2.4512E+07	6.3700E+05	6.1667E+07	6.3838E+06	1.5403E+08	5.5846E+06	5.4607E+06	3.7007E+07	1.3013E+08
	Ave	2.0427E+06	5.5394E+07	1.6121E+06	1.4331E+08	1.5356E+07	2.8334E+08	1.3490E+07	1.3241E+07	5.6950E+07	4.6608E+08
	Std	5.6058E+05	2.0532E+07	4.5499E+05	4.1531E+07	5.7444E+06	5.7190E+07	4.0886E+06	4.9062E+06	1.3062E+07	2.5347E+08
	Rank	2	6	1	8	5	9	4	3	7	10

**Table A4:** The experimental results on of HERIME and other competitors based on CEC2017 (100D).

No.	Metric	HERIME	RIME	EO	SAO	MRFO	IYDSE	DFDBARO	EOSMA	IGWO	LSHADE
F1	Best	2.5842E+07	7.1582E+08	2.3548E+08	3.7922E+10	7.0223E+09	3.5698E+10	6.3027E+07	1.3346E+09	5.6001E+09	2.8742E+10
	Ave	5.2503E+07	1.4881E+09	5.3995E+08	5.0081E+10	1.3042E+10	5.6883E+10	2.9057E+08	2.9102E+09	1.2800E+10	2.4043E+11
	Std	1.8256E+07	4.0307E+08	4.0689E+08	6.1049E+09	3.4348E+09	1.1324E+10	1.1216E+08	1.0495E+09	4.8878E+09	1.1454E+11
	Rank	1	4	3	8	7	9	2	5	6	10
F2	Best	2.8070E+05	2.7997E+05	2.0253E+05	2.7714E+05	2.4148E+05	2.5976E+05	3.1585E+05	2.2956E+05	2.3928E+05	7.6596E+05
	Ave	3.6962E+05	3.8774E+05	3.0153E+05	3.3042E+05	2.9018E+05	3.3526E+05	3.5291E+05	3.1193E+05	3.1524E+05	1.0330E+06
	Std	4.2812E+04	4.2934E+04	4.1326E+04	1.8510E+04	2.8075E+04	1.6175E+04	1.7520E+04	4.2845E+04	4.5457E+04	1.4591E+05
	Rank	8	9	2	5	1	6	7	3	4	10
F3	Best	6.8922E+02	9.6828E+02	8.8194E+02	4.4713E+03	1.5438E+03	5.6997E+03	1.1060E+03	1.0182E+03	1.1654E+03	6.1299E+03
	Ave	7.9222E+02	1.1210E+03	1.0160E+03	6.0480E+03	2.4774E+03	8.3892E+03	1.1908E+03	1.2825E+03	1.8842E+03	6.5873E+04
	Std	4.8979E+01	9.8447E+01	7.1978E+01	8.7376E+02	4.8380E+02	1.9992E+03	5.7443E+01	1.2362E+02	4.1159E+02	4.2495E+04
	Rank	1	3	2	8	7	9	4	5	6	10
F4	Best	7.1863E+02	9.5839E+02	9.1633E+02	1.5219E+03	1.2051E+03	1.5827E+03	1.1133E+03	9.7360E+02	8.5765E+02	1.5996E+03
	Ave	8.1223E+02	1.1074E+03	1.0394E+03	1.6443E+03	1.3618E+03	1.6918E+03	1.2630E+03	1.0929E+03	1.1186E+03	2.1370E+03
	Std	5.6509E+01	7.2916E+01	6.3633E+01	5.1085E+01	6.8618E+01	5.8648E+01	7.9036E+01	5.3123E+01	1.7684E+02	2.7277E+02
	Rank	1	4	2	8	7	9	6	3	5	10
F5	Best	6.0203E+02	6.2268E+02	6.0596E+02	6.4759E+02	6.4860E+02	6.8054E+02	6.2004E+02	6.1231E+02	6.1655E+02	6.3148E+02
	Ave	6.0419E+02	6.3377E+02	6.0951E+02	6.5495E+02	6.6659E+02	6.9145E+02	6.2717E+02	6.1866E+02	6.2346E+02	7.0679E+02
	Std	1.2706E+00	6.3416E+00	2.1169E+00	4.1954E+00	7.3355E+00	4.3813E+00	3.7414E+00	3.3790E+00	4.5006E+00	2.5394E+01
	Rank	1	6	2	7	8	9	5	3	4	10
F6	Best	1.2078E+03	1.6854E+03	1.3486E+03	2.1915E+03	2.3444E+03	2.2260E+03	1.4988E+03	1.4924E+03	1.4184E+03	2.0304E+03
	Ave	1.4074E+03	1.9261E+03	1.4729E+03	2.4050E+03	2.6867E+03	2.4772E+03	1.9314E+03	1.6597E+03	1.7336E+03	5.9785E+03
	Std	1.0343E+02	1.3022E+02	7.9867E+01	1.0584E+02	2.0730E+02	1.2308E+02	1.5497E+02	9.3522E+01	1.7346E+02	2.3496E+03
	Rank	1	5	2	7	9	8	6	3	4	10
F7	Best	1.0247E+03	1.2345E+03	1.2026E+03	1.8641E+03	1.5938E+03	1.8907E+03	1.4297E+03	1.2826E+03	1.2487E+03	1.8044E+03
	Ave	1.1121E+03	1.3938E+03	1.3203E+03	1.9375E+03	1.7540E+03	1.9856E+03	1.6164E+03	1.3876E+03	1.4449E+03	2.3736E+03
	Std	6.0430E+01	7.4700E+01	5.9303E+01	4.1290E+01	9.6160E+01	5.9457E+01	9.7475E+01	5.9326E+01	1.6100E+02	2.8533E+02
	Rank	1	4	2	8	7	9	6	3	5	10
F8	Best	1.0233E+03	1.3588E+04	5.1434E+03	2.1349E+04	3.3338E+04	4.9092E+04	2.2846E+04	7.2389E+03	1.2452E+04	2.6547E+04
	Ave	1.3611E+03	2.4023E+04	1.3208E+04	3.7471E+04	4.9537E+04	6.3458E+04	3.3368E+04	1.3918E+04	2.5110E+04	1.2505E+05
	Std	2.5265E+02	6.0521E+03	4.7351E+03	7.5945E+03	9.7412E+03	5.9122E+03	6.0074E+03	4.0648E+03	6.9445E+03	3.6434E+04
	Rank	1	4	2	7	8	9	6	3	5	10
F9	Best	1.4714E+04	1.6497E+04	1.5013E+04	2.9305E+04	1.7607E+04	2.8486E+04	1.3314E+04	2.0241E+04	1.4846E+04	3.0622E+04
	Ave	1.9874E+04	1.9473E+04	1.9876E+04	3.1168E+04	2.2017E+04	3.0026E+04	1.5808E+04	2.5397E+04	2.8710E+04	3.2491E+04
	Std	2.4010E+03	1.1610E+03	1.9750E+03	6.4908E+02	2.5640E+03	6.0866E+02	1.0070E+03	1.7785E+03	5.4260E+03	6.0413E+02
	Rank	3	2	4	9	5	8	1	6	7	10
F10	Best	2.4114E+03	6.8988E+03	6.7208E+03	6.0469E+04	4.5649E+04	1.1346E+05	5.3904E+04	1.6315E+04	5.5189E+03	2.8730E+05
	Ave	2.7368E+03	1.1190E+04	1.2323E+04	8.5979E+04	6.5737E+04	1.5732E+05	9.8651E+04	2.6609E+04	1.1494E+04	4.2852E+05
	Std	1.8389E+02	2.7200E+03	3.2851E+03	1.2663E+04	1.0321E+04	1.7809E+04	2.5531E+04	6.8866E+03	2.6544E+03	7.4243E+04
	Rank	1	2	4	7	6	9	8	5	3	10
F11	Best	2.8739E+07	3.0938E+08	4.2875E+07	8.3978E+09	3.7023E+08	8.3060E+09	2.1904E+08	8.3736E+07	7.7084E+08	2.0670E+10
	Ave	1.1132E+08	9.6791E+08	9.2472E+07	1.2479E+10	7.5079E+08	1.5541E+10	4.1769E+08	2.8855E+08	1.9751E+09	8.0778E+10
	Std	6.1357E+07	3.2237E+08	3.2453E+07	2.1663E+09	2.1898E+08	3.9524E+09	1.8084E+08	9.8559E+07	9.5689E+08	3.1825E+10
	Rank	2	6	1	8	5	9	4	3	7	10
F12	Best	4.5036E+04	1.3997E+06	1.7336E+04	7.4552E+08	2.1165E+05	8.4762E+08	1.0064E+05	4.2053E+04	3.4530E+06	3.4736E+06
	Ave	9.1195E+04	3.4906E+06	4.2041E+04	1.3347E+09	4.8803E+05	1.5615E+09	4.3300E+06	8.3991E+04	7.4917E+07	2.2420E+09
	Std	3.2221E+04	2.1298E+06	1.6704E+04	2.7261E+08	1.6661E+05	4.2730E+08	5.3508E+06	2.5806E+04	8.6736E+07	2.0588E+09
	Rank	3	5	1	8	4	9	6	2	7	10
F13	Best	3.1527E+03	1.2753E+06	5.3882E+05	3.5185E+06	7.0599E+05	2.7903E+06	1.5448E+06	3.8013E+05	5.2859E+05	4.5701E+07
	Ave	4.9264E+03	5.1075E+06	1.7466E+06	8.3408E+06	1.9466E+06	4.6075E+06	4.6207E+06	1.1655E+06	2.5691E+06	1.0020E+08
	Std	9.3389E+02	3.1623E+06	8.0166E+05	2.7605E+06	8.8604E+05	9.4375E+05	2.2032E+06	5.2919E+05	1.0919E+06	2.8496E+07
	Rank	1	8	3	9	4	6	7	2	5	10
F14	Best	1.0635E+04	2.1270E+05	4.0942E+03	1.1791E+08	1.5263E+04	1.2206E+08	6.9642E+03	1.3978E+04	1.2521E+06	4.9352E+06
	Ave	2.8204E+04	6.8130E+05	1.1221E+04	3.6384E+08	3.0149E+04	3.2478E+08	5.6802E+05	4.6700E+04	1.4169E+07	4.6125E+08
	Std	9.2649E+03	4.9306E+05	5.8907E+03	9.5108E+07	8.8715E+03	1.6229E+08	6.6956E+05	1.6806E+04	1.3000E+07	4.8059E+08
	Rank	2	6	1	9	3	8	5	4	7	10
F15	Best	4.4454E+03	4.6893E+03	3.9848E+03	9.8254E+03	4.6857E+03	9.3749E+03	4.8109E+03	4.1302E+03	4.5661E+03	1.0568E+04
	Ave	5.9649E+03	6.3766E+03	5.4861E+03	1.0928E+04	6.4198E+03	1.0415E+04	6.6713E+03	5.7404E+03	5.7915E+03	1.3720E+04
	Std	6.2362E+02	7.6566E+02	7.6718E+02	3.9085E+02	7.4077E+02	4.3315E+02	6.5090E+02	6.9675E+02	1.1400E+03	1.2156E+03
	Rank	4	5	1	9	6	8	7	2	3	10
F16	Best	3.4476E+03	4.0216E+03	3.4181E+03	7.5390E+03	3.9996E+03	7.1804E+03	5.1043E+03	3.7175E+03	3.5614E+03	8.6954E+03
	Ave	4.7805E+03	5.2620E+03	4.6920E+03	8.4062E+03	5.2236E+03	8.0113E+03	5.9816E+03	4.6611E+03	4.8080E+03	1.7440E+04
	Std	5.1982E+02	5.3158E+02	6.7377E+02	3.9350E+02	5.3448E+02	5.3162E+02	4.4417E+02	4.9672E+02	8.6537E+02	7.1614E+03
	Rank	3	6	2	9	5	8	7	1	4	10
F17	Best	5.9837E+04	7.6715E+05	1.0128E+06	8.1007E+06	7.2990E+05	2.7714E+06	1.0953E+06	7.4812E+05	7.2519E+05	4.7104E+07
	Ave	9.6003E+04	7.0102E+06	2.7924E+06	1.4888E+07	2.9812E+06	4.6861E+06	6.9449E+06	1.9994E+06	3.1500E+06	1.6361E+08
	Std	1.8371E+04	4.3033E+06	1.0005E+06	4.5006E+06	1.6718E+06	9.9805E+05	3.5097E+06	7.8663E+05	1.5749E+06	5.4809E+07
	Rank	1	8	3	9	4	6	7	2	5	10
F18	Best	6.2617E+03	2.7405E+06	2.4630E+03	1.8048E+08	1.5515E+04	1.4111E+08	2.4641E+04	1.8655E+04	4.1917E+06	1.7975E+06
	Ave	2.4396E+04	1.0537E+07	6.1336E+03	3.5570E+08	5.4681E+04	3.4853E+08	6.9430E+05	9.2380E+04	2.1129E+07	1.6378E+09
	Std	1.7927E+04	6.6824E+06	4.9204E+03	9.7905E+07	3.3726E+04	1.4807E+08	6.8752E+05	6.7104E+04	1.3060E+07	2.0361E+09
	Rank	2	6	1	9	3	8	5	4	7	10
F19	Best	4.0581E+03	4.5462E+03	3.4882E+03	6.1777E+03	4.1877E+03	6.2077E+03	4.6182E+03	3.8008E+03	4.1204E+03	7.4813E+03
	Ave	5.0465E+03	5.4178E+03	4.7911E+03	7.1059E+03	5.4924E+03	6.8723E+03	5.3606E+03	5.1360E+03	6.2625E+03	8.0442E+03
	Std	4.5976E+02	4.3808E+02	5.7514E+02	3.0825E+02	5.1920E+02	2.3181E+02	3.7086E+02	5.2877E+02	1.1147E+03	2.5350E+02
	Rank	2	5	1	9	6	8	4	3	7	10
F20	Best	2.5288E+03	2.7736E+03	2.6413E+03	3.3912E+03	2.9851E+03	3.3746E+03	3.0128E+03	2.7270E+03	2.7712E+03	3.3533E+03
	Ave	2.6495E+03	2.9376E+03	2.7622E+03	3.4521E+03	3.2248E+03	3.6155E+03	3.2150E+03	2.8549E+03	2.9237E+03	4.0588E+03
	Std	6.6105E+01	6.6372E+01	5.6179E+01	3.6395E+01	1.1054E+02	9.0598E+01	1.0778E+02	6.1723E+01	1.3368E+02	3.2983E+02
	Rank	1	5	2	8	7	9	6	3	4	10
F21	Best	1.7991E+04	1.9085E+04	1.8839E+04	7.1475E+03	2.0132E+04	3.0766E+04	1.6412E+04	2.1539E+04	1.9172E+04	3.3602E+04
	Ave	2.1652E+04	2.2135E+04	2.1962E+04	2.9259E+04	2.4328E+04	3.2593E+04	1.8127E+04	2.7766E+04	3.1469E+04	3.4650E+04
	Std	2.2953E+03	1.7184E+03	1.5985E+03	9.8027E+03	2.6585E+03	5.1428E+02	9.2372E+02	2.0190E+03	4.6631E+03	3.8163E+02
	Rank	2	4	3	7	5	9	1	6	8	10
F22	Best	3.0619E+03	3.2775E+03	3.0924E+03	3.9355E+03	3.7561E+03	4.0856E+03	3.3945E+03	3.2131E+03	3.2768E+03	3.7816E+03
	Ave	3.1500E+03	3.4184E+03	3.1844E+03	4.0419E+03	4.0351E+03	4.2365E+03	3.5980E+03	3.3481E+03	3.4449E+03	4.7173E+03
	Std	5.1304E+01	6.3851E+01	4.9455E+01	4.6227E+01	1.2599E+02	7.1549E+01	8.2397E+01	6.3970E+01	9.0999E+01	3.8369E+02
	Rank	1	4	2	8	7	9	6	3	5	10
F23	Best	3.4869E+03	3.7347E+03	3.5320E+03	4.4708E+03	4.5316E+03	4.9639E+03	4.1217E+03	3.6704E+03	3.7993E+03	4.5521E+03
	Ave	3.5985E+03	3.9513E+03	3.6424E+03	4.6251E+03	4.9885E+03	5.2511E+03	4.4660E+03	3.7914E+03	4.0087E+03	5.9234E+03
	Std	5.9012E+01	8.2356E+01	5.7302E+01	6.4300E+01	1.8895E+02	1.3447E+02	1.4776E+02	4.9535E+01	1.1275E+02	7.8845E+02
	Rank	1	4	2	7	8	9	6	3	5	10
F24	Best	3.3897E+03	3.6696E+03	3.5852E+03	5.9632E+03	4.2197E+03	6.5136E+03	3.6241E+03	3.7849E+03	4.1214E+03	6.7889E+03
	Ave	3.5270E+03	3.9050E+03	3.7482E+03	7.2435E+03	4.7752E+03	8.6824E+03	3.7787E+03	4.0463E+03	4.7014E+03	4.6111E+04
	Std	5.6936E+01	1.1078E+02	8.7348E+01	5.6404E+02	3.0504E+02	9.5123E+02	7.3972E+01	1.1169E+02	3.3386E+02	2.1210E+04
	Rank	1	4	2	8	7	9	3	5	6	10
F25	Best	8.0401E+03	1.1045E+04	8.6861E+03	1.7794E+04	1.1364E+04	2.0508E+04	1.6034E+04	9.9035E+03	1.1105E+04	1.9030E+04
	Ave	9.0037E+03	1.3183E+04	1.0080E+04	1.9240E+04	2.1132E+04	2.3830E+04	1.8477E+04	1.1453E+04	1.3182E+04	3.5314E+04
	Std	5.9418E+02	1.1383E+03	7.4797E+02	5.8322E+02	3.0221E+03	1.4863E+03	1.6252E+03	8.5214E+02	1.0947E+03	7.7113E+03
	Rank	1	5	2	7	8	9	6	3	4	10
F26	Best	3.3666E+03	3.5571E+03	3.4073E+03	4.0379E+03	3.9961E+03	4.5843E+03	3.7769E+03	3.5823E+03	3.6177E+03	3.2000E+03
	Ave	3.4534E+03	3.7839E+03	3.5020E+03	4.2958E+03	4.4290E+03	5.0163E+03	4.0611E+03	3.6970E+03	3.7855E+03	3.2000E+03
	Std	3.7288E+01	1.0655E+02	5.7907E+01	1.6563E+02	2.0199E+02	2.0575E+02	1.5852E+02	6.7212E+01	9.4917E+01	6.7264E-05
	Rank	2	5	3	8	9	10	7	4	6	1
F27	Best	3.5046E+03	3.8032E+03	3.7188E+03	7.0893E+03	4.6191E+03	8.8136E+03	3.7491E+03	4.0394E+03	4.3496E+03	3.3000E+03
	Ave	3.6383E+03	4.3775E+03	3.8997E+03	8.5390E+03	6.0742E+03	1.0611E+04	4.2551E+03	4.4558E+03	5.7398E+03	3.3000E+03
	Std	7.0914E+01	3.7547E+02	9.9766E+01	6.7698E+02	6.0720E+02	9.7206E+02	1.5567E+03	2.6547E+02	7.7867E+02	7.3039E-05
	Rank	2	5	3	9	8	10	4	6	7	1
F28	Best	5.4965E+03	6.8133E+03	5.1212E+03	1.0159E+04	7.0681E+03	1.1088E+04	7.2128E+03	5.2926E+03	6.1883E+03	1.1301E+04
	Ave	6.5841E+03	8.0523E+03	6.3753E+03	1.1511E+04	8.4953E+03	1.3327E+04	8.3607E+03	7.0759E+03	7.2256E+03	2.0810E+04
	Std	4.9628E+02	5.9988E+02	6.0321E+02	5.3194E+02	6.6111E+02	1.1373E+03	5.2700E+02	6.6360E+02	5.1637E+02	1.0109E+04
	Rank	2	5	1	8	7	9	6	3	4	10
F29	Best	4.7821E+05	3.9854E+07	8.1436E+04	6.3996E+08	2.4016E+06	7.6129E+08	2.3901E+06	1.5554E+06	5.3549E+07	6.5121E+06
	Ave	1.0004E+06	1.0791E+08	4.7573E+05	1.2117E+09	5.9925E+06	1.7027E+09	5.2405E+06	3.5763E+06	1.4511E+08	2.5067E+09
	Std	5.3738E+05	4.1278E+07	3.5853E+05	2.2334E+08	3.0380E+06	5.5394E+08	2.1087E+06	1.6193E+06	7.0118E+07	2.5961E+09
	Rank	2	6	1	8	5	9	4	3	7	10

**Table A5:** The experimental results on of HERIME and other competitors based on CEC2022 (10D).

No.	Metric	HERIME	RIME	EO	SAO	MRFO	IYDSE	DFDBARO	EOSMA	IGWO	LSHADE
F1	Best	3.0000E+02	3.1035E+02	3.0993E+02	4.0412E+02	5.7167E+02	2.1431E+03	1.6985E+03	3.6235E+02	3.0700E+02	6.7481E+03
	Ave	3.0003E+02	3.5250E+02	3.9348E+02	1.3688E+03	1.4395E+03	4.4170E+03	7.2592E+03	7.0741E+02	4.2243E+02	1.7123E+04
	Std	1.9162E-02	3.7965E+01	1.0219E+02	7.0725E+02	6.5070E+02	1.1714E+03	2.2746E+03	2.1775E+02	8.1059E+01	5.1370E+03
	Rank	1	2	3	6	7	8	9	5	4	10
F2	Best	4.0025E+02	4.0005E+02	4.0670E+02	4.0236E+02	4.0005E+02	4.4036E+02	4.0932E+02	4.0006E+02	4.0026E+02	4.0908E+02
	Ave	4.0780E+02	4.1683E+02	4.1251E+02	4.1093E+02	4.1571E+02	4.6915E+02	4.5135E+02	4.0504E+02	4.0592E+02	4.1529E+02
	Std	2.1555E+00	2.4875E+01	1.5403E+01	9.9852E+00	2.6916E+01	1.8547E+01	2.6255E+01	3.6800E+00	6.6188E+00	4.9360E+00
	Rank	3	8	5	4	7	10	9	1	2	6
F3	Best	6.0002E+02	6.0032E+02	6.0000E+02	6.0083E+02	6.0046E+02	6.1658E+02	6.0071E+02	6.0011E+02	6.0041E+02	6.0105E+02
	Ave	6.0007E+02	6.0120E+02	6.0002E+02	6.0204E+02	6.0149E+02	6.2741E+02	6.0609E+02	6.0035E+02	6.0100E+02	6.0525E+02
	Std	3.8082E-02	6.9845E-01	2.7802E-02	5.3256E-01	1.1861E+00	6.9942E+00	2.9786E+00	1.6909E-01	5.4783E-01	2.8778E+00
	Rank	2	5	1	7	6	10	9	3	4	8
F4	Best	8.0403E+02	8.0706E+02	8.0310E+02	8.1054E+02	8.0318E+02	8.2294E+02	8.1785E+02	8.0458E+02	8.0493E+02	8.3141E+02
	Ave	8.1352E+02	8.2106E+02	8.0962E+02	8.3174E+02	8.2008E+02	8.3630E+02	8.3287E+02	8.1258E+02	8.2631E+02	8.4528E+02
	Std	6.3491E+00	9.0548E+00	4.3113E+00	9.2091E+00	8.1485E+00	5.2980E+00	7.4731E+00	4.7825E+00	1.0673E+01	5.5929E+00
	Rank	3	5	1	7	4	9	8	2	6	10
F5	Best	9.0000E+02	9.0015E+02	9.0000E+02	9.0044E+02	9.0021E+02	9.5310E+02	9.3778E+02	9.0000E+02	9.0019E+02	9.1218E+02
	Ave	9.0002E+02	9.0246E+02	9.0009E+02	9.0129E+02	9.0965E+02	1.0929E+03	9.9096E+02	9.0043E+02	9.0112E+02	9.9642E+02
	Std	6.5876E-02	3.7303E+00	1.8429E-01	8.1102E-01	3.1758E+01	9.6058E+01	3.0155E+01	6.2121E-01	1.0874E+00	6.6690E+01
	Rank	1	6	2	5	7	10	8	3	4	9
F6	Best	1.8015E+03	2.0799E+03	1.8705E+03	3.3954E+03	2.0745E+03	1.4954E+04	2.3557E+03	2.2097E+03	2.9411E+03	2.5275E+04
	Ave	1.8082E+03	6.6853E+03	4.6564E+03	3.2327E+04	5.0436E+03	2.6283E+05	5.6028E+03	6.4713E+03	1.8084E+04	2.7765E+05
	Std	8.8723E+00	4.5087E+03	2.2532E+03	3.1534E+04	4.1562E+03	2.1834E+05	1.9316E+03	3.7278E+03	1.4610E+04	2.4769E+05
	Rank	1	6	2	8	3	9	4	5	7	10
F7	Best	2.0009E+03	2.0032E+03	2.0010E+03	2.0206E+03	2.0071E+03	2.0462E+03	2.0140E+03	2.0100E+03	2.0050E+03	2.0276E+03
	Ave	2.0213E+03	2.0220E+03	2.0209E+03	2.0321E+03	2.0304E+03	2.0673E+03	2.0243E+03	2.0235E+03	2.0301E+03	2.0389E+03
	Std	5.7057E+00	6.3227E+00	4.6390E+00	4.6551E+00	7.7434E+00	1.0714E+01	4.0201E+00	5.7397E+00	7.9886E+00	5.0769E+00
	Rank	2	3	1	8	7	10	5	4	6	9
F8	Best	2.2011E+03	2.2052E+03	2.2032E+03	2.2152E+03	2.2127E+03	2.2267E+03	2.2131E+03	2.2106E+03	2.2094E+03	2.2257E+03
	Ave	2.2192E+03	2.2234E+03	2.2222E+03	2.2281E+03	2.2292E+03	2.2324E+03	2.2236E+03	2.2244E+03	2.2269E+03	2.2307E+03
	Std	7.1496E+00	4.0703E+00	4.8399E+00	4.2942E+00	3.3958E+00	2.6847E+00	1.8651E+00	4.7610E+00	5.0396E+00	2.2709E+00
	Rank	1	3	2	7	8	10	4	5	6	9
F9	Best	2.5293E+03	2.5293E+03	2.5293E+03	2.5294E+03	2.5309E+03	2.5546E+03	2.5325E+03	2.5293E+03	2.5296E+03	2.4877E+03
	Ave	2.5293E+03	2.5294E+03	2.5293E+03	2.5301E+03	2.5358E+03	2.5996E+03	2.5679E+03	2.5295E+03	2.5312E+03	2.4950E+03
	Std	2.1169E-04	1.6949E-01	2.0838E-04	2.0547E+00	5.0622E+00	2.3755E+01	4.2121E+01	1.5004E-01	4.9709E+00	4.8244E+00
	Rank	3	4	2	6	8	10	9	5	7	1
F10	Best	2.5002E+03	2.5003E+03	2.5002E+03	2.5004E+03	2.5004E+03	2.5013E+03	2.4809E+03	2.5003E+03	2.5003E+03	2.5005E+03
	Ave	2.5069E+03	2.5224E+03	2.5301E+03	2.5132E+03	2.5178E+03	2.5071E+03	2.5055E+03	2.5004E+03	2.5240E+03	2.5099E+03
	Std	2.6394E+01	4.7853E+01	4.8818E+01	3.8694E+01	4.1370E+01	4.0061E+00	1.1594E+01	6.5383E-02	4.8381E+01	7.6000E+00
	Rank	3	8	10	6	7	4	2	1	9	5
F11	Best	2.6019E+03	2.6930E+03	2.6001E+03	2.6268E+03	2.6125E+03	2.7657E+03	2.7862E+03	2.6080E+03	2.6143E+03	2.8556E+03
	Ave	2.8745E+03	2.9625E+03	2.7790E+03	2.7065E+03	2.7148E+03	2.9239E+03	2.9024E+03	2.7407E+03	2.9007E+03	3.3036E+03
	Std	8.9985E+01	1.9549E+02	1.4500E+02	8.7326E+01	1.4334E+02	1.7897E+02	1.1750E+02	1.3050E+02	1.0297E+02	2.1065E+02
	Rank	5	9	4	1	2	8	7	3	6	10
F12	Best	2.8614E+03	2.8629E+03	2.8614E+03	2.8609E+03	2.8652E+03	2.8645E+03	2.8738E+03	2.8597E+03	2.8629E+03	2.8487E+03
	Ave	2.8637E+03	2.8652E+03	2.8634E+03	2.8638E+03	2.8705E+03	2.8702E+03	2.8853E+03	2.8641E+03	2.8650E+03	2.8620E+03
	Std	8.4843E-01	1.2241E+00	1.0292E+00	1.0970E+00	4.9891E+00	1.9384E+00	9.1987E+00	1.4211E+00	1.1537E+00	8.8844E+00
	Rank	3	7	2	4	9	8	10	5	6	1

**Table A6:** The experimental results on of HERIME and other competitors based on CEC2022 (20D).

No.	Metric	HERIME	RIME	EO	SAO	MRFO	IYDSE	DFDBARO	EOSMA	IGWO	LSHADE
F1	Best	3.0051E+02	1.1260E+03	1.2366E+03	9.3597E+03	8.8432E+03	2.3073E+04	1.9173E+04	3.3310E+03	1.5190E+03	2.4741E+04
	Ave	3.0329E+02	3.2302E+03	4.4912E+03	1.8022E+04	1.6186E+04	3.3395E+04	3.1726E+04	7.4538E+03	3.7269E+03	6.5920E+04
	Std	1.9698E+00	1.5598E+03	2.4360E+03	4.8277E+03	4.5058E+03	5.3703E+03	6.2457E+03	2.2407E+03	1.4062E+03	1.4255E+04
	Rank	1	2	4	7	6	9	8	5	3	10
F2	Best	4.4492E+02	4.3053E+02	4.2995E+02	4.6018E+02	4.5204E+02	5.0185E+02	4.7571E+02	4.4933E+02	4.4823E+02	4.3220E+02
	Ave	4.4886E+02	4.6375E+02	4.5657E+02	4.7792E+02	4.8517E+02	6.4066E+02	5.8675E+02	4.6212E+02	4.7898E+02	5.3335E+02
	Std	9.9228E-01	2.0824E+01	1.1289E+01	1.0577E+01	2.6681E+01	5.6332E+01	4.7479E+01	9.6681E+00	2.8555E+01	9.2784E+01
	Rank	1	4	2	5	7	10	9	3	6	8
F3	Best	6.0004E+02	6.0087E+02	6.0008E+02	6.0632E+02	6.0281E+02	6.3743E+02	6.0432E+02	6.0057E+02	6.0157E+02	6.0445E+02
	Ave	6.0017E+02	6.0413E+02	6.0022E+02	6.1188E+02	6.1020E+02	6.5230E+02	6.1177E+02	6.0149E+02	6.0337E+02	6.2170E+02
	Std	7.2220E-02	1.9919E+00	1.3976E-01	2.3267E+00	7.2140E+00	8.7635E+00	5.5449E+00	5.8322E-01	1.6975E+00	1.0656E+01
	Rank	1	5	2	8	6	10	7	3	4	9
F4	Best	8.0822E+02	8.2609E+02	8.1262E+02	8.9087E+02	8.2603E+02	8.7895E+02	8.5567E+02	8.1899E+02	8.2066E+02	9.0450E+02
	Ave	8.2810E+02	8.5788E+02	8.3211E+02	9.2320E+02	8.6353E+02	9.2740E+02	8.9522E+02	8.4613E+02	8.7275E+02	9.4374E+02
	Std	1.1636E+01	2.2913E+01	1.1171E+01	9.7215E+00	1.7124E+01	1.4640E+01	2.0217E+01	1.0799E+01	3.6048E+01	1.5341E+01
	Rank	1	4	2	8	5	9	7	3	6	10
F5	Best	9.0002E+02	9.0184E+02	9.0010E+02	9.5696E+02	9.1536E+02	1.5477E+03	1.2129E+03	9.0070E+02	9.0213E+02	1.0616E+03
	Ave	9.0018E+02	1.0918E+03	9.0199E+02	1.0273E+03	1.5731E+03	2.2932E+03	1.7069E+03	9.0817E+02	9.5342E+02	2.3956E+03
	Std	1.6829E-01	2.9294E+02	2.5847E+00	4.9664E+01	5.3618E+02	3.6292E+02	2.9154E+02	6.7811E+00	5.3550E+01	7.3289E+02
	Rank	1	6	2	5	7	9	8	3	4	10
F6	Best	1.8531E+03	1.0197E+04	2.0155E+03	3.7279E+05	4.0660E+03	2.7111E+06	9.4460E+03	3.1929E+03	7.6770E+03	3.9241E+07
	Ave	1.9426E+03	1.0475E+05	4.1341E+03	4.8912E+06	2.3206E+04	1.7257E+07	1.8835E+05	4.0467E+04	9.9709E+04	1.1849E+08
	Std	9.2276E+01	9.4489E+04	2.6993E+03	4.5209E+06	1.5547E+04	8.9272E+06	2.0987E+05	8.9709E+04	1.0331E+05	5.9499E+07
	Rank	1	6	2	8	3	9	7	4	5	10
F7	Best	2.0251E+03	2.0293E+03	2.0262E+03	2.0600E+03	2.0402E+03	2.1194E+03	2.0424E+03	2.0314E+03	2.0347E+03	2.1049E+03
	Ave	2.0424E+03	2.0655E+03	2.0429E+03	2.0965E+03	2.0913E+03	2.1547E+03	2.0960E+03	2.0548E+03	2.0589E+03	2.1518E+03
	Std	1.3858E+01	2.0641E+01	1.4482E+01	1.7687E+01	2.9228E+01	1.7357E+01	2.8566E+01	1.2035E+01	1.8971E+01	2.0763E+01
	Rank	1	5	2	8	6	10	7	3	4	9
F8	Best	2.2240E+03	2.2256E+03	2.2241E+03	2.2329E+03	2.2283E+03	2.2322E+03	2.2262E+03	2.2278E+03	2.2271E+03	2.2447E+03
	Ave	2.2275E+03	2.2375E+03	2.2292E+03	2.2446E+03	2.2381E+03	2.2465E+03	2.2401E+03	2.2327E+03	2.2364E+03	2.2736E+03
	Std	1.9848E+00	2.1964E+01	3.4447E+00	4.9092E+00	1.6233E+01	6.7695E+00	1.1702E+01	2.5755E+00	4.0313E+00	1.2857E+01
	Rank	1	5	2	8	6	9	7	3	4	10
F9	Best	2.4808E+03	2.4812E+03	2.4808E+03	2.4841E+03	2.4832E+03	2.5383E+03	2.4850E+03	2.4810E+03	2.4814E+03	2.4705E+03
	Ave	2.4808E+03	2.4831E+03	2.4808E+03	2.4891E+03	2.4906E+03	2.5905E+03	2.4940E+03	2.4819E+03	2.4862E+03	2.4958E+03
	Std	1.4228E-02	1.2508E+00	4.3133E-02	3.5071E+00	5.1956E+00	2.0539E+01	6.6575E+00	5.8172E-01	5.1487E+00	1.5082E+01
	Rank	1	4	2	6	7	10	8	3	5	9
F10	Best	2.4146E+03	2.5005E+03	2.5003E+03	2.5008E+03	2.5006E+03	2.5255E+03	2.4430E+03	2.5004E+03	2.5005E+03	2.5822E+03
	Ave	2.5631E+03	2.6464E+03	2.9952E+03	2.7125E+03	2.5427E+03	3.0345E+03	2.5529E+03	2.5007E+03	2.6041E+03	4.2100E+03
	Std	1.3549E+02	2.5308E+02	6.6814E+02	6.9709E+02	2.0740E+02	8.6477E+02	6.3913E+01	7.7194E-01	4.4824E+02	1.2718E+03
	Rank	4	6	8	7	2	9	3	1	5	10
F11	Best	2.9127E+03	3.0964E+03	2.9015E+03	3.3975E+03	2.7758E+03	3.9770E+03	3.0215E+03	2.9777E+03	3.0411E+03	4.9467E+03
	Ave	2.9362E+03	3.3653E+03	2.9059E+03	3.5578E+03	3.0816E+03	4.9217E+03	3.3625E+03	3.0324E+03	3.3842E+03	1.0758E+04
	Std	9.9379E+00	1.1479E+02	2.4801E+00	7.6368E+01	1.3237E+02	5.0752E+02	1.0472E+02	3.8285E+01	1.9587E+02	5.4730E+03
	Rank	2	6	1	8	4	9	5	3	7	10
F12	Best	2.9320E+03	2.9370E+03	2.9359E+03	2.9495E+03	2.9790E+03	2.9882E+03	2.9864E+03	2.9393E+03	2.9405E+03	2.9000E+03
	Ave	2.9417E+03	2.9571E+03	2.9434E+03	2.9607E+03	3.0138E+03	3.0393E+03	3.0570E+03	2.9524E+03	2.9572E+03	2.9000E+03
	Std	4.8671E+00	1.5002E+01	4.9191E+00	8.3073E+00	2.6268E+01	2.2512E+01	4.4040E+01	6.6908E+00	9.3202E+00	5.4884E-05
	Rank	2	5	3	7	8	9	10	4	6	1
